# A German multicenter real‐world analysis of talquetamab in 138 patients with relapsed/refractory multiple myeloma

**DOI:** 10.1002/hem3.70114

**Published:** 2025-04-17

**Authors:** Jan H. Frenking, Christine Riedhammer, Raphael Teipel, Florian Bassermann, Britta Besemer, Moritz Bewarder, Jan Braune, Annamaria Brioli, Franziska Brunner, Maria Dampmann, Roland Fenk, Deniz N. Gezer, Sarah Goldman‐Mazur, Christine Hanoun, Marion Högner, Cyrus Khandanpour, Katja Kolditz, Igor Kos, Jan Krönke, Miriam Kull, Valentine Landrin, Theo Leitner, Maximilian Merz, Ivana von Metzler, Christian S. Michel, Carsten Müller‐Tidow, Sebastian Theurich, Karolin Trautmann‐Grill, Ralph Wäsch, Romans Zukovs, Mathias Hänel, Leo Rasche, Marc S. Raab

**Affiliations:** ^1^ Heidelberg Myeloma Center, Department of Medicine V University Hospital and Medical Faculty Heidelberg Heidelberg University Heidelberg Germany; ^2^ Clinical Cooperation Unit Molecular Hematology/Oncology German Cancer Research Center (DKFZ) Heidelberg Germany; ^3^ Department of Internal Medicine II University Hospital of Würzburg Würzburg Germany; ^4^ Department of Internal Medicine I University Hospital Carl Gustav Carus Dresden Dresden Germany; ^5^ Internal Medicine III TUM University Hospital Technical University of Munich München Germany; ^6^ TranslaTUM Center for Translational Cancer Research Technical University of Munich München Germany; ^7^ Bavarian Cancer Research Center (BZKF) Germany; ^8^ Deutsches Konsortium für Translationale Krebsforschung (DKTK) Heidelberg Germany; ^9^ Internal Medicine II University Hospital Tübingen Tübingen Germany; ^10^ Department of Internal Medicine I Saarland University Medical Center Homburg Germany; ^11^ Department of Hematology, Oncology and Cancer Immunology Charité – Berlin University Medicine Berlin Germany; ^12^ Internal Medicine C, Hematology, Oncology, Stem Cell Transplantation and Palliative Care University Medicine Greifswald Greifswald Germany; ^13^ Department of Hematology, Hemostasis, Oncology, and Stem Cell Transplantation Hannover Medical School Hannover Germany; ^14^ Department of Internal Medicine IV University Hospital Halle Halle Germany; ^15^ Department of Hematology and Stem Cell Transplantation University Hospital Essen Essen Germany; ^16^ Department of Haematology, Oncology and Clinical Immunology University Hospital of Düsseldorf Düsseldorf Germany; ^17^ Department of Hematology, Oncology, Hemostaseology and Stem Cell Transplantation University Hospital Aachen Aachen Germany; ^18^ Department of Hematology, Cellular Therapy, Hemostaseology and Infectious Diseases University of Leipzig Medical Center Leipzig Germany; ^19^ Department of Haematology and Oncology University Medical Center Schleswig‐Holstein University Cancer Center and University of Lübeck Lübeck Germany; ^20^ Department of Internal Medicine III Klinikum Chemnitz Chemnitz Germany; ^21^ Department of Internal Medicine III University Hospital Ulm Ulm Germany; ^22^ Department of Internal Medicine II Frankfurt University Hospital Frankfurt Germany; ^23^ Department of Internal Medicine III University Medical Center Mainz Mainz Germany; ^24^ National Center for Tumor Diseases (NCT) Heidelberg Germany; ^25^ Department of Medicine III LMU University Hospital Munich München Germany; ^26^ Department of Hematology, Oncology and Stem Cell Transplantation Medical Center – University of Freiburg Faculty of Medicine, University of Freiburg Freiburg Germany

## Abstract

Bispecific T‐cell engagers (BTCEs) represent a paradigm shift in the treatment of relapsed/refractory multiple myeloma (RRMM). Talquetamab, a GPRC5DxCD3 BTCE, has shown promising results in the MonumenTAL‐1 trial and was recently approved by the Food and Drug Administration and the European Medicines Agency. However, treatment under real‐world conditions may not represent patient characteristics in clinical trials with restricted enrollment criteria. We performed a retrospective real‐world analysis including 138 RRMM patients treated with talquetamab at 21 German centers. Of evaluable patients, 43% had ISS stage III, 37% had extraosseous disease, and 48% had high‐risk cytogenetics. After a median of six prior therapy lines, 58% of patients would not have been eligible for MonumenTAL‐1. With a median follow‐up of 8.2 months, we observed an overall response rate of 65% and a median progression‐free survival of 6.4 months (95% confidence interval 5.1–9.0). Prior BTCE exposure, ISS stage III, extraosseous disease, and penta‐drug refractory disease were associated with unfavorable outcomes. Grade ≥ 3 cytokine release syndrome and neurotoxicity occurred in 5.1% and 1.5% of patients, respectively. In summary, our real‐world study confirms the efficacy and safety of talquetamab, despite a high proportion of patient‐ and disease‐related risk factors. These results support its use as bridging or long‐term treatment, even in advanced stages.

## Graphical abstract

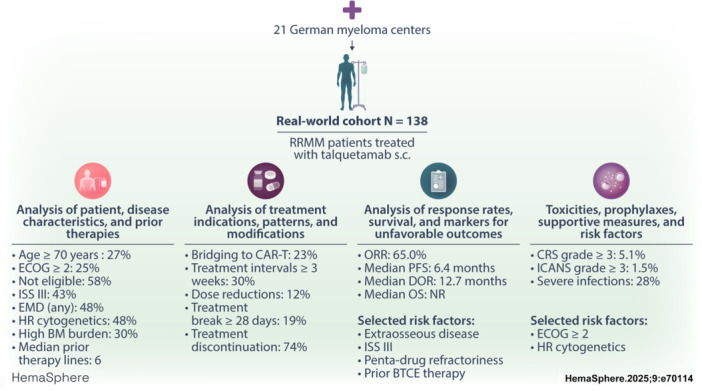

## INTRODUCTION

Bispecific T‐cell engagers (BTCEs) are reshaping the treatment landscape for relapsed/refractory multiple myeloma (RRMM) and are increasingly being investigated in early disease stages and in combination regimens in clinical trials.^1^, ^2^ The BTCE talquetamab induces an immune synapse by binding CD3 on T cells and G protein‐coupled receptor of family C, group 5, member D (GPRC5D) on myeloma cells, leading to T cell activation, degranulation, and tumor cell lysis.^3^, ^4^, ^5^ In the pivotal multicenter, open label, phase 1 MonumenTAL‐1 trial, talquetamab has demonstrated an adequate safety profile while eliciting deep and durable responses in heavily pretreated RRMM patients.^6^ For the two recommended phase 2 doses of subcutaneous talquetamab, 405 μg/kg weekly and 800 μg/kg every other week, Chari et al. reported an overall response of 70% and 64% and a median duration of response (DOR) of 10.2 and 7.8 months, respectively. Cytokine release syndrome (CRS) occurred in 77% and 80% of patients, respectively, with one grade 3 event. Neurotoxic events were reported at a frequency of 10% and 5%, respectively, without grade ≥ 3 events.^6^ As a result, talquetamab was recently approved by the Food and Drug Administration (FDA) and the European Medicines Agency (EMA) for RRMM patients with four and three prior lines of therapy, respectively, including an immunomodulatory agent, a proteasome inhibitor, and an anti‐CD38 antibody.

Clinical trials are considered the gold standard for evaluating novel therapies. However, real‐world analyses have emerged as a complementary source of evidence, particularly in the field of T‐cell redirecting immunotherapies,^7^, ^8^, ^9^ and provide first‐of‐its‐kind data on patient populations typically excluded from trials. Moreover, this development is based on discrepancies between trial and standard treatment outcomes in the past, which can be attributed to several factors.^9^, ^10^ Trial patients must meet strict enrollment criteria, usually have superior socio‐economic resources, and benefit from intensive clinical monitoring and specific treatment instructions. However, the recent German real‐world analysis of teclistamab has demonstrated an efficacy and safety profile comparable to the results of the MajesTEC‐1 trial, although almost half of the patients would not have been eligible.^9^, ^11^

So far, reports on real‐world experiences with talquetamab are limited, and a comparison with the MonumenTAL‐1 trial has not yet been conducted. In addition, practical expertise with talquetamab is growing, and clinicians explore individualized treatment concepts, indications, and strategies for toxicity management and prophylaxis, leading to the need for systematic evaluations and reliable markers to identify patients at risk for adverse outcomes. We aimed to address these knowledge gaps by performing a retrospective, multicenter real‐world analysis of the efficacy and safety of talquetamab in RRMM and to summarize common treatment patterns across institutions in Germany.

## METHODS

### Patient selection and data collection

**Figure 1:**
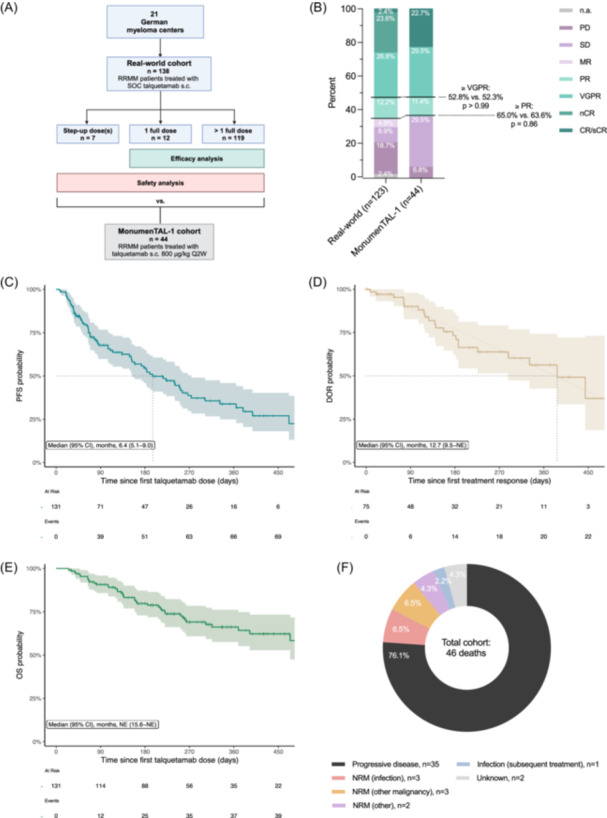
**Efficacy of talquetamab in the real‐world setting. (A)** Cohort description and analysis workflow. The total real‐world cohort included 138 relapsed/refractory multiple myeloma (RRMM) patients who had received standard‐of‐care (SOC) talquetamab at one of 21 German centers. The safety analyses included all patients with available data, unless otherwise stated. The efficacy analyses included all evaluable patients who had received ≥1 full dose of talquetamab. Details on treatment application and status and loss to follow‐up are provided in Table S1. Patient and disease characteristics, safety, and efficacy were compared to the MonumenTAL‐1 study cohort of patients treated with subcutaneous talquetamab at a dose of 800 μg/kg every other week. **(B)** Comparison of response rates between the real‐world cohort and the MonumenTAL‐1 cohort. Kaplan–Meier estimates of **(C)** progression‐free survival (PFS), **(D)** duration of response (DOR), and **(E)** overall survival (OS). The median survival time in months and the 95% confidence interval (CI), the number of evaluable patients at risk and the number of events are provided below the curve. The light shading of the curve indicates the 95% confidence intervals. **(F)** Causes of a total of 46 deaths. The non‐relapse mortality (NRM) cases included three patients who died from sepsis, three patients who died from other malignancies or related complications, one patient who died from an esophageal rupture, and one patient who died from Stevens‐Johnson syndrome/toxic epidermal necrolysis. Another case of NRM due to an infection occurred after subsequent CAR T‐cell therapy and was therefore listed separately. CI, confidence interval; CR, complete response; DOR, duration of response; MR, minimal response; n.a., not available; nCR, near complete response; NE, not estimable; NRM, non‐relapse mortality; OS, overall survival; PD, progressive disease; PFS, progression‐free survival; PR, partial response; RRMM, relapsed/refractory multiple myeloma; s.c., subcutaneous; sCR, stringent complete response; SD, stable disease; SOC, standard of care; VGPR, very good partial response.

This multicenter retrospective observational study included 138 RRMM patients who had received standard‐of‐care (SOC) talquetamab at 21 German myeloma centers until November 2024 (Figure 1A). Data cut‐off was January 6, 2025. The median follow‐up time for the total cohort was 7.8 months (95% confidence interval [CI] 6.8–8.7). For efficacy analyses, only patients with ≥1 full dose were considered. All other analyses including safety assessments referred to the total cohort, unless otherwise stated. All patients with available data were included in the respective evaluations. Clinical data were extracted from the electronic patient management software and the original internal or external medical records whenever available. Missing data were mainly due to external care with limited or without data access and consecutive loss to follow‐up (Table S1). Study results were reported according to the STROBE guidelines.^12^

### Treatment

Treatment with talquetamab, modifications of doses or intervals, and toxicity management were conducted according to the specialist information and the institutional standard operating procedures, with individual adjustments at the physician's discretion. Most patients (*n* = 104/138; 75%) had received the approved 800 μg/kg dose administered subcutaneously every 2 weeks at treatment initiation and/or on the last treatment day, after step‐up dosing with 10, 60, and 400 μg/kg. Further information on the intended and applied treatment regimens, doses, interruptions, and discontinuations is provided in Table S1 and the “Results” section.

### Definitions, classifications, and grading systems

Treatment response was determined by the investigators according to the International Myeloma Working Group (IMWG) criteria,^13^ with the usual adjustments for retrospective studies.^7^, ^9^ Near complete response (nCR) was defined as serological complete response (CR) without available bone marrow (BM) status. Imaging studies and confirmatory tests were not mandatory for response assessment. Patients without response data were summarized under the category *not available*. Patients with no measurable disease and no evidence of disease progression were classified as *not evaluable* and excluded from further response analyses. (Revised) International Staging System (ISS/R‐ISS) stage before the first talquetamab dose,^14^, ^15^ treatment refractoriness, prior lines of therapy, and event endpoints^16^, ^17^ were defined according to international consensus. Extramedullary disease (EMD) manifestations before the start of treatment were classified as bone‐associated (paramedullary), bone‐independent, organ‐infiltrating, or plasma cell leukemia.^18^ Extraosseous disease included the latter three types or combinations. High‐risk cytogenetics included del(17p), t(4;14), and/or t(14;16)^6^, ^9^ in the most recent test available. Chromosome 1q21 abnormalities (+1q) were specified as 1q gain (three copies) or amplification (≥4 copies).^19^, ^20^ CRS and immune effector cell‐associated neurotoxicity syndrome (ICANS) were graded as recommended by the American Society for Transplantation and Cellular Therapy (ASTCT).^21^ Other adverse events were graded according to the Common Terminology Criteria for Adverse Events (CTCAE) v5.0. Grade ≥ 3 events were defined as severe. Cytopenias were graded according to the lowest reported value for each cell lineage. Infections were recorded on the basis of clinical, microbiological, and radiological findings. Only infection events occurring after the first full dose of talquetamab were considered to facilitate the differential diagnosis of CRS, unless otherwise stated. Opportunistic infections were defined according to the literature.^22^, ^23^ The step‐up dosing period comprised the days between the first step‐up dose and the first full dose. Cycles referred to the subsequent 28‐day treatment periods. Non‐relapse mortality was defined as death without prior disease progression.^24^

### Comparison with the MonumenTAL‐1 trial

Patient and disease characteristics, efficacy, and safety were compared to the results of the MonumenTAL‐1 trial published by Chari et al.^6^ As most patients in our cohort had received the approved bi‐weekly administration, the comparison was restricted to trial patients treated with subcutaneous talquetamab at a dose of 800 μg/kg every other week (*n* = 44). Eligibility was assessed according to the inclusion and exclusion criteria in the original study protocol published by Chari et al.,^6^ taking into account the later amendment for patients with prior allogeneic stem cell transplantation.

### Statistical analysis

R (v4.4.1), GraphPad Prism (v10.2.3), Microsoft Excel (v16.87), and BioRender were used for statistical data analysis and visualization. Non‐parametric Mann–Whitney test was used to compare continuous variables. Percentages were compared with Fisher's exact test. Uni‐ or multivariate logistic regression analyses were performed to examine associations between treatment response, grade ≥ 2 CRS, any grade ICANS, and selected binary or continuous variables. The group of treatment responders included patients with a partial response (PR) or better. Non‐responders included patients with a minimal response or worse and patients with unavailable responses. Associations with progression‐free survival (PFS), DOR, and overall survival (OS) were analyzed by uni‐ or multivariate Cox proportional hazard regression models. Multivariate models were based on a priori selected variables taking into account established risk factors in the literature on T‐cell redirecting immunotherapies,^6^, ^7^, ^9^, ^11^ data availability, and event numbers. Kaplan–Meier curves were used to estimate PFS, DOR, and OS. Survival curves were compared by the log‐rank test. The observation periods for response and safety analyses ended if a new or additional treatment was started, and patients without prior disease progression were censored in these cases. All *p* values were two‐sided and considered statistically significant at *p* < 0.05.

## RESULTS

### Treatment application and status

Of the 138 patients, seven (5%) had received only step‐up doses, 12 (9%) had received only one full dose of the intended regimen, and 119 (86%) had received more than one full dose (Figure 1A). The 800 μg/kg Q2W scheme was the most frequently intended and applied regimen (Table S1). The cohort with more than one full dose (*n* = 119) included four patients (3%) with weekly treatment, 79 patients (66%) with bi‐weekly treatment, five patients (4%) with a switch to 3‐week intervals, and 31 patients (26%) with a switch to ≥4‐week intervals, mostly initiated between cycle 4 and 6 (*n* = 12/31; 39%) or earlier (*n* = 11/31; 35%). Side effects were the most common reason for extending the intervals beyond 2 weeks (*n* = 29/36; 81%). Reductions in the absolute treatment dose were observed in 12% of patients (*n* = 16/132). Talquetamab was intended until progression in 105 of the 138 patients (76%) and used as bridging therapy to another treatment in 33 patients (24%), including 18 patients with a treatment start before and 14 patients with a start after T‐cell apheresis for chimeric antigen receptor (CAR) T‐cell manufacturing. At data cut‐off, 102 out of 137 patients (74%) with available data had discontinued treatment. Further details are provided in Table S1.

### Patient and disease characteristics

Baseline characteristics determined before treatment initiation and a comparison with the MonumenTAL‐1 cohort are shown in Table 1. The median age was 64 years (range 24–84). Of the patients with available data, 25% (*n* = 33/133) had an ECOG ≥ 2, 43% (*n* = 49/114) had ISS stage III, and 48% (*n* = 60/124) had EMD manifestations, including 37% (*n* = 46/124) with extraosseous disease. Moreover, 48% (*n* = 57/120) had high‐risk cytogenetics based on the latest available findings. Patients had received a median of six prior therapy lines (range 2–15). Triple‐class and penta‐drug refractory disease were found in 86% (*n* = 118/138) and 47% (*n* = 64/136) of patients, respectively. Of patients, 20% (*n* = 28/138) had received prior BTCE therapy (mostly teclistamab), and 34% (*n* = 47/138) had received prior CAR T‐cell therapy (mostly idecabtagene vicleucel). Baseline laboratory parameters and cytopenias were determined immediately before the first dose and are listed in Table S2. Of note, 47% of patients (*n* = 65/138) showed elevated lactate dehydrogenase (LDH) levels, and a significant proportion of patients had CTC grade ≥ 3 cytopenias before treatment initiation.

**Table 1:** Patient and disease characteristics.

	Real‐world cohort, *n* = 138	MonumenTAL‐1 cohort,^a^ *n* = 44	*p* value
Age, years			
Median (range)	64 (24–84)	64 (47–84)	
<70, no./total no. (%)	101/138 (73)	29/44 (66)	0.35
≥70, no./total no. (%)	37/138 (27)	15/44 (34)	
Sex, no./total no. (%)			
Male	96/138 (70)	21/44 (48)	**0.01**
Female	42/138 (30)	23/44 (52)	
ECOG,^b^ no./total no. (%)			
0–1	100/133 (75)		
2–3	33/133 (25)		
Time from initial diagnosis to first talquetamab dose, years, median (range)	6.4 (0.6–24.4)	6.4 (0.8–21.3)	
Myeloma subtype,^b^ no./total no. (%)			
Intact immunoglobulin	77/138 (56)		
Light chain	48/138 (35)		
Hypo‐/Asecretory	13/138 (9)		
ISS stage,^b^ no./total no. (%)			
I	34/114 (30)	16/43 (37)	**0.03**
II	31/114 (27)	18/43 (42)	
III	49/114 (43)	9/43 (21)	
R‐ISS stage,^b^ no./total no. (%)			
I	10/110 (9)		
II	65/110 (59)		
III	35/110 (32)		
Extramedullary disease,^b^ no./total no. (%)			
None	64/124 (52)	29/44 (66)	0.11
Any^c^	60/124 (48)	15/44 (34)	
Bone‐associated (paramedullary)	14/124 (11)		
Bone‐independent/organ‐infiltrating	28/124 (23)		
Both	15/124 (12)		
Plasma cell leukemia (±other)	3/124 (2)		
Extraosseous	46/124 (37)		
Cytogenetics,^d^ no./total no. (%)			
Standard risk	63/120 (53)	31/40 (78)	**0.006**
High risk	57/120 (48)	9/40 (23)	
del(17p)	32/123 (26)	7/40 (18)	
t(4;14)	21/120 (18)	3/40 (8)	
t(14;16)	10/120 (8)	0/40 (0)	
Others			
+1q	59^e^/113 (52)		
t(14;20)	1/116 (1)		
Bone marrow burden,^b^ no./total no. (%)			
<60%	35/50 (70)	36/41 (88)	**0.05**
≥60%	15/50 (30)	5/41 (12)	
Prior therapies			
Prior therapy lines, median (range)	6 (2–15)	5 (2–17)	
Autologous SCT, no./total no. (%)	119/138 (86)	33/44 (75)	0.10
Allogeneic SCT, no./total no. (%)	16/138 (12)		
BCMA‐targeted therapy,^f^ no./total no. (%)	71/138 (51)	12/44 (27)	**0.006**
Antibody‐drug conjugate	10/138 (7)		
GPRC5D‐targeted therapy, no./total no. (%)	0/138 (0)		
T‐cell redirecting immunotherapy,^g^ no./total no. (%)	66/138 (48)		
Bispecific T‐cell engager, no./total no. (%)	28/138 (20)		
Teclistamab	25/138 (18)		
Elranatamab	2/138 (1)		
Other (BCMA‐directed)	1/138 (1)		
Time from last application to first talquetamab dose, months, median (IQR)	1.9 (0.8–5.9), *n* = 27		
CAR T‐cell therapy, no./total no. (%)	47/138 (34)		
Idecabtagene vicleucel	44/138 (32)		
Ciltacabtagene autoleucel	2/138 (1)		
Other (SLAMF7‐directed)	1/138 (1)		
Time from last application to first talquetamab dose, months, median (IQR)	11.4 (7.6–14.2), *n* = 46		
Classical cytotoxic chemotherapy ≤ 60 days before first talquetamab dose, no./total no. (%)	33/136 (24)		
Classical cytotoxic polychemotherapy^h^ ≤ 60 days before first talquetamab dose, no./total no. (%)	15/138 (11)		
Time from last systemic therapy to first talquetamab dose, months, median (IQR)	1.1 (0.7–3.9), *n* = 127		
Treatment status, no./total no. (%)			
IMiD^i^ refractory	131/138 (95)	42/44 (95)	>0.99
PI^j^ refractory	130/138 (94)	36/44 (82)	**0.03**
Anti‐CD38^k^ refractory	135/138 (98)	39/44 (89)	**0.02**
Triple‐class refractory^l^	118/138 (86)	33/44 (75)	0.11
Penta‐drug exposed^m^	112/138 (81)	30/44 (68)	0.09
Penta‐drug refractory^m^	64/136 (47)	9/44 (20)	**0.002**

*Note:* Significant *p*‐values are printed in bold.

Abbreviations: BCMA, B‐cell maturation antigen; CAR, chimeric antigen receptor; CI, confidence interval; ECOG, Eastern Cooperative Oncology Group performance status; GPRC5D, G protein‐coupled receptor, family C, group 5, member D; IMiD, immunomodulatory agent; IQR, interquartile range; ISS, International Staging System; PI, proteasome inhibitor; R‐ISS, Revised International Staging System; SCT, stem cell transplant; SLAMF7, SLAM family member 7.

^a^ Patients who had received subcutaneous talquetamab 800 μg/kg every 2 weeks (Chari et al., 2022).

^b^ Determined before the first dose of talquetamab (for real‐world cohort).

^c^ Extramedullary disease included all listed disease manifestations. In contrast, extraosseous disease was defined as the presence of bone‐independent/organ‐infiltrating disease manifestations or plasma cell leukemia (±other extramedullary disease manifestations). Only bone‐associated (paramedullary) disease manifestations were not classified as extraosseous disease.

^d^ Based on the latest available cytogenetic findings.

^e^ Gain (3 copies): *n* = 38/113 (34%); amplification (≥4 copies): *n* = 8/113 (7%); not specified: *n* = 13/113 (12%).

^f^ Including antibody‐drug conjugates, bispecific antibodies, and CAR T‐cell therapy.

^g^ Including bispecific antibodies and CAR T‐cell therapy.

^h^ Combination of ≥2 classical cytotoxic agents.

^i^ Immunomodulatory agents included thalidomide, lenalidomide, or pomalidomide.

^j^ Proteasome inhibitors included bortezomib, carfilzomib, or ixazomib.

^k^ Anti‐CD38 monoclonal antibodies included daratumumab or isatuximab.

^l^ Refractory to at least one immunomodulatory agent, at least one proteasome inhibitor, and at least one anti‐CD38 monoclonal antibody.

^m^ Exposed/Refractory to at least two proteasome inhibitors, at least two immunomodulatory agents, and at least one anti‐CD38 monoclonal antibody.

### Eligibility

Of 134 patients with available data, 78 patients (58%) would not have met the original eligibility criteria of the MonumenTAL‐1 trial evaluated at the day of informed consent and listed in Table S3, including 34 patients (25%) with ≥2 exclusion criteria. Among the common reasons for non‐eligibility were thrombocytopenia (*n* = 30/134; 22%), renal dysfunction (*n* = 20/134; 15%), application of selected antitumor therapies within prohibited time periods before talquetamab initiation (*n* = 18; 13%), and anemia (*n* = 17/134; 13%).

### CRS and ICANS

A detailed overview of treatment complications, supportive, and prophylactic measures is given in Table 2. Most patients were admitted as inpatients for step‐up dosing (*n* = 132/138; 96%), with a median duration of hospitalization of 12 days (interquartile range [IQR] 10–16). Ninety‐six out of 137 evaluable patients (70%) were affected by CRS, including six grade 3 events (4%) and one grade 4 event (1%). ICANS occurred in 9% of evaluable patients (*n* = 12/137), with one grade 3 (1%) and one grade 4 event (1%). Compared to the trial cohort, we observed a lower rate of tocilizumab administrations (35% vs. 55%; *p* = 0.03) and an increased use of steroids (20% vs. 7%; *p* = 0.06). Of note, one patient experienced grade 3 CRS and grade 4 ICANS treated with anakinra and noradrenaline as additional supportive measures.

**Table 2:** Safety, supportive measures, and prophylaxes.

	Real‐world cohort *n* = 138			MonumenTAL‐1 cohort^a^ *n* = 44	*p* value
Step‐up dosing					
Outpatient, no./total no. (%)	6/138 (4)				
Inpatient, no./total no. (%)	132/138 (96)				
Days, median (IQR)	12 (10–16), *n* = 130				
Intravenous antibiotics, no./total no. (%)	86/135 (64)				
Days, median (IQR)	6 (0–9), *n* = 135				
**CRS**,^b^ no./total no. (%)					
None	41/137 (30)			9/44 (20)	0.25
Any	96/137 (70)			35/44 (80)	
Grade 1	65/137 (47)				
Grade 2	24/137 (18)				
Grade 3	6/137 (4)				
Grade 4	1/137 (1)				
Neurotoxicity,^b^ no./total no. (%)					
None	125/137 (91)			42 (95)	0.52
Any	12/137 (9)			2 (5)	
Grade 1	6/137 (4)				
Grade 2	4/137 (3)				
Grade 3	1/137 (1)				
Grade 4	1/137 (1)				
Supportive measures, no./total no. (%)					
Tocilizumab	48/137 (35)			24/44 (55)	**0.03**
Steroids	27/137 (20)			3/44 (7)	0.06
Anakinra	1/137 (1)				
Cytopenias, no./total no. (%)	Step‐up/Cycle 1^c^	Subsequent cycles	Overall	Overall	*p* value
Anemia					
None	1/137 (1)	3/99 (3)	0/138 (0)	25/44 (57)	**<0.0001**
Any	136/137 (99)	96/99 (97)	138/138 (100)	19/44 (43)	
Grade 1	39/137 (28)	42/99 (42)	33/138 (24)	9/44 (20)	
Grade 2	40/137 (29)	36/99 (36)	43/138 (31)		
Grade ≥ 3	57/137 (42)	18/99 (18)	62/138 (45)	10/44 (23)	
Thrombocytopenia					
None	24/137 (18)	31/98 (32)	22/138 (16)	34/44 (77)	**<0.0001**
Any	113/137 (82)	67/98 (68)	116/138 (84)	10/44 (23)	
Grade 1	38/137 (28)	37/98 (38)	39/138 (28)	5/44 (11)	
Grade 2	23/137 (17)	9/98 (9)	24/138 (17)		
Grade 3	18/137 (13)	11/98 (11)	17/138 (12)	5/44 (11)	
Grade 4	34/137 (25)	10/98 (10)	36/138 (26)		
Leukopenia					
None	24/136 (18)	40/100 (40)	22/138 (16)	36/44 (82)	**<0.0001**
Any	112/136 (82)	60/100 (60)	116/138 (84)	8/44 (18)	
Grade 1	21/136 (15)	16/100 (16)	20/138 (14)	2/44 (5)	
Grade 2	42/136 (31)	31/100 (31)	41/138 (30)		
Grade 3	32/136 (24)	11/100 (11)	37/138 (27)	6/44 (14)	
Grade 4	17/136 (13)	2/100 (2)	18/138 (13)		
Neutropenia					
None	53/121 (44)	52/93 (56)	54/132 (41)	28/44 (64)	**0.002**
Any	68/121 (56)	41/93 (44)	78/132 (59)	16/44 (36)	
Grade 1	12/121 (10)	15/93 (16)	12/132 (9)	2/44 (5)	
Grade 2	20/121 (17)	11/93 (12)	23/132 (17)		
Grade 3	20/121 (17)	10/93 (11)	24/132 (18)	14/44 (32)	
Grade 4	16/121 (13)	5/93 (5)	19/132 (14)		
Supportive measures, no./total no. (%)					
G‐CSF	18/138 (13)				
TPO agonists	2/138 (1)				
RBC transfusion	56/138 (41)				
PLT transfusion	30/138 (22)				
Infections,^d^ no./total no. (%)					
Any	80/131 (61)			15/44 (34)	**0.003**
Severe (grade ≥ 3)	37/131 (28)			3/44 (7)	**0.003**
Viral	7/131 (5)				
Bacterial	10/131 (8)				
Fungal	1/131 (1)				
>1 pathogen	6/131 (5)				
No pathogen identified	13/131 (10)				
Supportive measures, no./total no. (%)					
IVIG substitution	69/138 (50)				
Prophylaxes, no./total no. (%)					
PCP prophylaxis	128/138 (93)				
Herpesvirus prophylaxis	138/138 (100)				
Hepatitis prophylaxis	3/138 (2)				
Antibiotic prophylaxis	5/138 (4)				
Antifungal prophylaxis	3/138 (2)				
Dysgeusia, no./total no. (%)					
None	36/136 (26)			19/44 (43)	0.04
Any	100/136 (74)			25/44 (57)	
Grade 1	19/136 (14)				
Grade 2,^e^ no loss of taste	17/136 (13)				
Grade 2,^e^ loss of taste	63/136 (46)				
Not specified	1/136 (1)				
Weight loss,^f^ no./total no. (%)					
None	55/121 (45)			30/44 (68)	**0.01**
Any	66/121 (55)			14/44 (32)	
Grade 1	46/121 (38)				
Grade 2	18/121 (15)				
Grade 3	2/121 (2)				
Intensive care unit treatment, no./total no. (%)	10/137 (7)				

*Note*: Significant *p*‐values are printed in bold.

Abbreviations: CRS, cytokine release syndrome. CTCAE, common terminology criteria for adverse events. G‐CSF, granulocyte‐colony stimulating factor. ICANS, immune effector cell‐associated neurotoxicity syndrome. IQR, interquartile range. IVIG, intravenous immunoglobulin. PCP, pneumocystis pneumonia. PLT transfusion, platelet transfusion. RBC transfusion, red blood cell transfusion. TPO agonists, thrombopoietin receptor agonists.

^a^ Patients who had received subcutaneous talquetamab 800 μg/kg every two weeks (Chari et al., 2022).

^b^ For the real‐world cohort, CRS and neurotoxicity were reported and graded according to the ASTCT CRS and ICANS consensus grading (see methods section).

^c^ Including the step‐up dosing period and the 28‐day period after the first full dose.

^d^ For the real‐world cohort, only infections after the first full dose of talquetamab were reported (see methods section).

^e^ Grade 2 according to CTCAE is defined as altered taste with change in diet, noxious or unpleasant taste or loss of taste. Patients with the latter type of dysgeusia are listed separately.

^f^ Distribution of weight groups based on body mass index prior to the first dose of talquetamab: underweight *n* = 2/136 (1%), normal *n* = 65/136 (48%), overweight *n* = 45/136 (33%), obesity *n* = 24/136 (18%).

### Characteristics associated with grade ≥ 2 CRS and any grade ICANS

Univariate logistic regression analysis was performed to examine associations between patient and disease characteristics and grade ≥ 2 CRS (Table S4). Patients who would have been eligible for MonumenTAL‐1 showed an inferior risk for grade ≥ 2 CRS (OR 0.33; *p* = 0.02). In contrast, baseline ECOG ≥ 2 (OR 3.34; *p* = 0.007), high‐risk cytogenetics (OR 2.94; *p* = 0.02), ISS stage III (OR 2.83; *p* = 0.03), and R‐ISS stage III (OR 2.86; *p* = 0.03) were associated with an increased risk. In a multivariate analysis (Table S5), ECOG ≥ 2 (OR 3.63; *p* = 0.04) and high‐risk cytogenetics (OR 4.49; *p* = 0.02) were predictive of grade ≥ 2 events. Due to limited event numbers, any grade ICANS was evaluated by univariate analysis only (Table S6). Patients with ECOG ≥ 2 (OR 4.38; *p* = 0.02), ISS stage III (OR 14.8; *p* = 0.01), or CRS grade ≥ 2 (OR 5.05; *p* = 0.01) were more likely to experience neurotoxicity.

### Cytopenias

By the end of cycle 1, CTC grade ≥ 3 anemia, thrombocytopenia, leukopenia, and neutropenia were reported with a frequency of 42% (*n* = 57/137), 38% (*n* = 52/137), 36% (*n* = 49/136), and 30% (*n* = 36/121), respectively (Table 2). During the subsequent cycles, grade ≥ 3 cytopenias were less common and observed in 18% (*n* = 18/99), 21% (*n* = 21/98), 13% (*n* = 13/100), and 16% (*n* = 15/93) of patients, respectively. Compared to the trial, we observed significant differences in the frequency and severity of cytopenias. A relevant proportion of patients required supportive measures, including growth factors (*n* = 18/138; 13%), and red blood cell (*n* = 56/138; 41%) and platelet transfusions (*n* = 30/138; 22%). Two patients (1%) received thrombopoietin receptor agonists.

### Infections and prophylaxes

At data cut‐off, infections of any grade after the first full dose were reported in 61% (*n* = 80/131) of evaluable patients (Table 2). Twenty‐eight percent (*n* = 37/131) were affected by a severe infection, including seven cases (5%) of a viral infection, 10 cases (8%) of a bacterial infection, and one case (1%) of a fungal infection. A severe bacterial and viral infection was reported in five patients (4%). One patient (1%) developed all three infection types. Opportunistic infections occurred in 21% (*n* = 27/131) of evaluable patients and were mostly caused by viral pathogens (*n* = 15/131; 11%) (Table S7).

Intravenous immunoglobulin substitution was given to 50% (*n* = 69/138) of patients due to low IgG levels, acute infections, a general infection tendency, or as primary prophylaxis in 64% (*n* = 44/69), 28% (*n* = 19/69), 23% (*n* = 16/69), and 19% (*n* = 13/69) of cases, respectively. All patients received a herpesvirus prophylaxis (mostly aciclovir), and 93% (*n* = 128/138) received pneumocystis pneumonia prophylaxis (mostly co‐trimoxazole).

### Other adverse events

Among the most common adverse events was dysgeusia (*n* = 100/136; 74%), with 63 patients (46%) reporting a loss of taste (Table 2). During treatment, the sense of taste worsened, remained constant, improved, or completely recovered in 2% (*n* = 2/84), 70% (*n* = 59/84), 24% (*n* = 20/84), and 4% (*n* = 3/84) of patients with available data, respectively. More than half of the evaluable patients (*n* = 66/121; 55%) were affected by weight loss, including 18 cases (15%) of CTC grade 2 and two cases (2%) of grade 3. Treatment breaks > 28 days were documented in 26 out of 137 patients (19%) with available data. Infections (*n* = 12/26; 46%), skin‐/nail‐related events (*n* = 7/26; 27%), and dysgeusia (*n* = 6/26; 23%) were given as common reasons (Table S1). The adverse events related to the treatment break recovered or resolved in 68% (*n* = 17/25) of affected patients. During the observation period, 7% (*n* = 10/137) of the examined patients required intensive care unit treatment for any reason.

### Efficacy

For efficacy analysis, we included all patients who had received ≥1 full dose of talquetamab (*n* = 131) (Figure 1A). The median follow‐up time for this group was 8.2 months (95% CI 6.9–9.4). Eight patients were classified as *not evaluable* and excluded from further response analyses as described in the “Methods” section. Among the remaining 123 patients, the overall response rate (≥PR) was 65% (*n* = 80/123) (Figure 1B and Table S8). Thirty‐two patients achieved an nCR or better (26%). Response rates were comparable between the real‐world and the MonumenTAL‐1 cohorts (Figure 1B and Table S8). The median time to the first and best response was 1.0 months (range 0.2–5.7) and 1.8 months (range 0.4–12.1), respectively. Interestingly, four patients showed increasing parameters of disease activity in the first response assessment, followed by a delayed response to treatment and possibly reflective of a tumor flare phenomenon. The median PFS was 6.4 months (95% CI 5.1–9.0) (Figure 1C), the median DOR was 12.7 months (95% CI 9.5 to not estimable [NE]) (Figure 1D), and the median OS was not reached (Figure 1E). The causes of death in the total cohort are illustrated in Figure 1F.

### Characteristics associated with inferior outcomes

**Figure 2:**
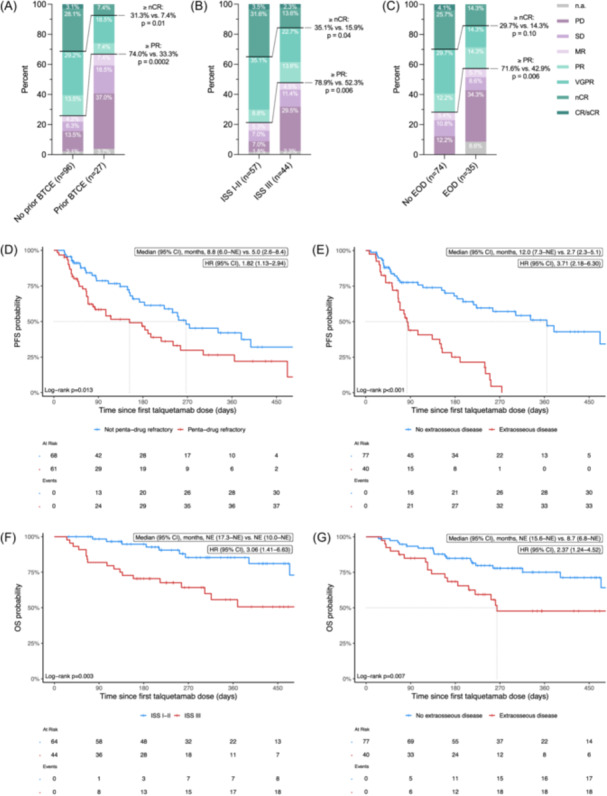
**Baseline characteristics associated with treatment response, progression‐free survival (PFS), and overall survival (OS)**. Comparison of response rates between patients with no prior bispecific T‐cell engager (BTCE) therapy and patients with prior BTCE therapy **(A)**, between patients with International Staging System (ISS) stages I and II and patients with ISS stage III before the first talquetamab dose **(B)**, and between patients with no extraosseous disease (EOD) and with EOD before the first talquetamab dose **(C)**. **(D)** Kaplan–Meier estimates of the probability of PFS for patients with no penta‐drug refractory disease (blue) and patients with penta‐drug refractory disease (red) (3‐month PFS 79% vs. 58%; 6‐month PFS 64% vs. 47%). **(E)** Kaplan–Meier estimates of the probability of PFS for patients with no EOD manifestations (blue) and patients with EOD manifestations (red) (3‐month PFS 78% vs. 44%; 6‐month PFS 68% vs. 25%). **(F)** Kaplan–Meier estimates of the probability of OS for patients with ISS stages I and II (blue) and patients with ISS stage III (red) (3‐month OS 98% vs. 82%; 6‐month OS 95% vs. 70%). **(G)** Kaplan–Meier estimates of the probability of OS for patients with no EOD manifestations (blue) and patients with EOD manifestations (red) (3‐month OS 93% vs. 85%; 6‐month OS 85% vs. 68%). The median survival times in months and the 95% confidence intervals (CI), the results of the Cox regression analysis (hazard ratio [HR] and 95% CI) and the log‐rank test, the number of evaluable patients at risk and the number of events are provided above or below the curves. BTCE, bispecific T‐cell engager; CI, confidence interval; CR, complete response; EOD, extraosseous disease; HR, hazard ratio; ISS, International Staging System; MR, minimal response; n.a., not available; nCR, near complete response; NE, not estimable; OS, overall survival; PD, progressive disease; PFS, progression‐free survival; PR, partial response; sCR, stringent complete response; SD, stable disease; VGPR, very good partial response.

Finally, we aimed to determine factors associated with inferior outcomes. In univariate analysis of treatment response (≥PR) (Table S9), extraosseous disease (OR 0.30; *p* = 0.005), ISS stage III (OR 0.29; *p* = 0.006), increased LDH (OR 0.44; *p* = 0.03), penta‐drug refractoriness (OR 0.26; *p* = 0.001), and prior BTCE therapy (OR 0.18; *p* < 0.001) were among the unfavorable factors. In a multivariate model (Table 3), prior BTCE therapy, ISS III, and extraosseous disease remained significantly associated with a decreased response rate. A trend toward significance was observed for penta‐drug refractoriness. The corresponding overall response rates are shown in Figure 2A–C.

**Table 3:** Multivariate analyses of treatment response, progression‐free survival, and overall survival.

	**Treatment response** a			**Progression‐free survival**			**Overall survival**		
**Variable**	**OR**	**95% CI**	***p* value**	**HR**	**95% CI**	***p* value**	**HR**	**95% CI**	***p* value**
Age [cont.]	1.04	0.98–1.11	0.17	0.96	0.93–0.99	**0.023**	0.99	0.95–1.03	0.58
ECOG ≥ 2^b^	0.60	0.10–4.03	0.58	0.97	0.36–2.61	0.96	3.32	0.99–11.2	0.052
Extraosseous disease^b^,^c^	0.16	0.03–0.72	**0.023**	9.49	3.90–23.1	**<0.001**	2.19	0.89–5.42	0.09
High‐risk cytogenetics^d^	0.56	0.11–2.62	0.47	1.42	0.66–3.03	0.37	0.74	0.26–2.08	0.56
ISS stage III^b^	0.10	0.02–0.44	**0.005**	1.69	0.79–3.63	0.18	2.37	0.93–6.01	0.07
Increased LDH (>ULN)^b^	0.72	0.17–3.03	0.65	1.60	0.76–3.37	0.22	2.14	0.77–5.91	0.14
Penta‐drug refractoriness^e^	0.22	0.04–0.94	0.053	2.91	1.21–6.95	**0.017**	1.23	0.42–3.59	0.70
Prior BTCE therapy	0.04	0.00–0.24	**0.001**	1.59	0.60–4.23	0.35	1.37	0.38–4.92	0.63

*Note*: Significant *p*‐values are printed in bold.

Abbreviations: BTCE, bispecific T‐cell engager; CI, confidence interval; ECOG, Eastern Cooperative Oncology Group performance status; HR, hazard ratio; ISS, International Staging System; LDH, lactate dehydrogenase; OR, odds ratio; ULN, upper limit of normal.

^a^ Best overall response ≥ partial response according to IMWG criteria (compared to patients with minimal response, stable disease, progressive disease, or unavailable response).

^b^ Determined before the first dose of talquetamab.

^c^ Extraosseous disease was defined as the presence of bone‐independent/organ‐infiltrating disease manifestations or plasma cell leukemia (±other extramedullary disease manifestations). Only bone‐associated (paramedullary) disease manifestations were not classified as extraosseous disease.

^d^ Based on the latest available cytogenetic findings.

^e^ Refractory to at least two proteasome inhibitors, at least two immunomodulatory agents, and at least one anti‐CD38 monoclonal antibody.

Regarding PFS, the univariate analysis showed similar, prognostically negative associations for the mentioned factors extraosseous disease (HR 3.71; *p* < 0.001), increased LDH (HR 2.17; *p* = 0.001), penta‐drug refractoriness (HR 1.82; *p* = 0.02), and prior BTCE exposure (HR 1.87; *p* = 0.02) (Table S10). In addition, patients with ECOG ≥ 2 (HR 1.72; *p* = 0.05), multiple (>6) prior lines of therapy (HR 1.75; *p* = 0.02), recent cytotoxic chemotherapy (HR 1.84; *p* = 0.02), and ICANS (HR 2.99; *p* = 0.007) demonstrated an inferior PFS. Of the mentioned variables, only extraosseous disease (HR 12.3; *p* < 0.001) and elevated LDH (HR 3.15; *p* = 0.009) were significantly associated with an adverse DOR (Table S11). In the multivariate model, age, extraosseous disease, and penta‐drug refractory disease were identified as independent predictors of PFS (Table 3 and Figure 2D,E).

Interestingly, a longer interval from the last BTCE exposure appeared to be beneficial in the subgroup analyses of patients with prior BTCE therapy (Tables S9 and S10 and Figure S1). No associations with prior CAR T‐cell therapy and the investigated subgroups were found (Tables S9 and S10 and Figure S2).

Patients with ECOG ≥ 2 (HR 2.41; *p* = 0.008), extraosseous disease (HR 2.37; *p* = 0.009), ISS stage III (HR 3.06; *p* = 0.005), high LDH (HR 2.62; *p* = 0.003), multiple prior therapies (HR 1.94; *p* = 0.04), prior BTCE therapy (HR 2.01; *p* = 0.04), and ICANS (HR 8.22; *p* < 0.001) showed an inferior OS in the univariate analysis (Table S12). In the multivariate model, ECOG ≥ 2, extraosseous disease, and ISS III showed a trend toward significance (Table 3 and Figure 2F,G).

## DISCUSSION

T‐cell redirecting immunotherapies such as BTCEs and CAR T cells are currently revolutionizing the treatment of multiple myeloma. Promising results in clinical trials,^6^, ^11^, ^25^ “off‐the‐shelf” availability, and toxicity‐adapted dosing of BTCEs favor their development. In recent months, accumulating evidence has confirmed the potential of teclistamab in RRMM,^8^, ^9^ while real‐world data on talquetamab is still limited. In this study, we performed a large multicenter retrospective analysis of patients treated with talquetamab in clinical routine to assess efficacy and tolerability in a real‐world setting.

Despite a high proportion of difficult‐to‐treat patients and the fact that the majority would not have been eligible for MonumenTAL‐1, we observed an overall response rate of 65.0%. The response rates in the MonumenTAL‐1 800 μg/kg cohort and the MajesTEC‐1 trial were 63.6%^6^ and 63.0%,^11^ respectively. Among the variables associated with inferior outcomes were extramedullary/extraosseous disease and ISS stage III, which represent established risk factors in multiple myeloma in general^14^, ^18^, ^26^ and also in the context of T‐cell redirecting immunotherapies.^6^, ^9^, ^11^, ^27^, ^28^ Moreover, patients with prior BTCE exposure showed inferior response rates, PFS, and OS compared to all others, while no differences in DOR were observed. A possible explanation could be T‐cell exhaustion related to prior (long‐term) therapy with teclistamab and consecutive treatment refractoriness when re‐exposed to another BTCE. The link between the pre‐existing T‐cell repertoire and initial treatment response to BTCEs has been well described by Friedrich et al.^29^ Moreover, a recent spatial transcriptomic analysis of EMD manifestations provided first insights into the distribution and exhaustion state of intralesional T cells as a resistance mechanism to BTCE therapy.^30^

In our real‐world cohort, any grade CRS and ICANS were observed with a frequency of 70% and 8.8%, respectively. Among patients treated with bi‐weekly subcutaneous talquetamab at the 800 μg/kg dose in the MonumenTAL‐1 trial, CRS and neurotoxicity rates were 80% and 4.5%, respectively.^6^ In the cohort treated with a weekly subcutaneous dose of 405 μg/kg, the rates were 77% and 10%, respectively. No grade ≥ 3 CRS events were reported for the 800 μg/kg cohort, and only one patient (3.3%) was affected at the 405 μg/kg dose level. Of note, no grade ≥ 3 neurotoxicity occurred with subcutaneous dosing.^6^ In contrast, our study showed severe CRS and ICANS rates of 5.1% and 1.5%, respectively. A comparison of CRS and neurotoxicity across studies has to be interpreted under consideration of potential differences in the applied grading systems (2014 criteria of Lee et al.^31^ or ASTCT criteria^21^). However, a trend toward more severe CRS and ICANS events in the real‐world setting has also been reported for teclistamab (3.6% and 4.5%, respectively).^8^ The extent of these toxicities depends on disease and treatment type, patient characteristics, baseline inflammation, and disease burden.^32^, ^33^, ^34^ In a real‐world analysis of idecabtagene vicleucel, Hansen et al. described associations between grade ≥ 3 CRS and ECOG ≥ 2, higher R‐ISS stage, and high BM burden.^7^ Grade ≥ 2 neurotoxicity was found to be associated with ECOG ≥ 2, elevated beta‐2 microglobulin, and ferritin levels.^7^ These findings are in line with the risk factors described in our analysis, comprising indicators of poor general condition (e.g., ECOG ≥ 2) and pronounced disease aggressiveness (e.g., high‐risk cytogenetics and ISS stage III), rates of which were higher in our cohort as compared to the clinical trial. Discrepancies regarding advanced CRS and ICANS between real‐world and clinical trial cohorts are therefore most likely explained by differences in patient and disease characteristics. Another explanation could be different administration strategies for tocilizumab. Only 35% of patients had received tocilizumab in our cohort, compared to 55% in the 800 μg/kg MonumenTAL‐1 cohort. Of note, several studies have shown the advantages of early tocilizumab to mitigate the risk for CRS aggravation and duration after CAR T‐cell therapy.^35^, ^36^, ^37^

The rates of all‐grade infections and severe infections in our study were 61% and 28%, respectively. In the MonumenTAL‐1 trial, the infection rates were 34% and 6.8% for the bi‐weekly 800 μg/kg dose and 47% and 6.7% for the weekly 405 μg/kg dose, respectively.^6^ Different frequencies must be interpreted under consideration of the respective follow‐up periods and might be explained by differences in supportive and prophylactic measures, a higher rate of patients with aggressive disease manifestations or comorbidities, and more severe cytopenias in the real‐world cohort. The latter observation must be seen in the context of the increased rate of pre‐existing cytopenias at baseline, which is an exclusion criterion for clinical trial enrollment and indicates a reduced BM reserve due to disease infiltration or intensive pretreatment.

Key limitations of our study are the retrospective design and less coherent dose reduction strategies and management of side effects, which are inherent to all real‐world analyses. The comparisons with the MonumenTAL‐1 trial should be interpreted with caution, particularly due to the differences in follow‐up time between the real‐world and trial cohorts (7.8 months [8.2 months in the efficacy cohort] and 4.2 months, respectively) and are not intended for clinical guidance. However, the combined analysis of 138 patients from 21 independent centers is a strength of our analysis and increases generalizability.

Future real‐world studies should investigate talquetamab in large international cohorts. Moreover, the identification of prognostic and predictive factors for outcomes and toxicities is critical to improve risk stratification and guide clinical decision‐making. Scores such as the CAR‐HEMATOTOX^38^ or EASIX^39^ have been demonstrated to identify vulnerable RRMM patients before anti‐B‐cell maturation antigen (BCMA) CAR T‐cell therapy and might also be helpful in the context of BTCE therapy.^40^, ^41^

In summary, our real‐world study confirms the efficacy and safety of talquetamab as a bridging or long‐term therapy in RRMM, even in advanced disease stages. Prophylaxes, supportive measures, and possibilities of dose adjustments should be fully utilized to reduce complications and improve quality of life throughout treatment. Patients with the aforementioned risk factors could benefit from more intensive clinical surveillance.

## AUTHOR CONTRIBUTIONS

Conceptualization: Jan H. Frenking, Marc S. Raab, Christine Riedhammer, and Leo Rasche. Methodology: Jan H. Frenking. Investigation: All authors. Analysis and visualization: Jan H. Frenking. Project administration: Jan H. Frenking and Marc S. Raab. Supervision: Marc S. Raab. Writing—original draft: Jan H. Frenking. Writing—review and editing: All authors. Guarantor: Marc S. Raab. All authors read and approved the final manuscript.

## CONFLICT OF INTEREST STATEMENT

J. H. F. declares an advisory role for Pfizer and has received honoraria from BMS and Stemline and travel and congress participation grants from Janssen‐Cilag. C. R. has received honoraria from Janssen‐Cilag. F. Ba. has received honoraria and/or travel/accommodation expenses from BMS, AbbVie, and Janssen. B. B. has received honoraria from Janssen‐Cilag, GSK, Amgen, Sanofi, Takeda, Pfizer, and Oncopeptides. M. B. has received honoraria from Janssen, GSK, and AstraZeneca. A. B. has participated in advisory boards from BMS, Janssen, GSK, Sanofi, AstraZeneca, and Menarini; received honoraria from Menarini; and received honoraria and travel support from BMS, Janssen, GSK, Sanofi, AstraZeneca, Amgen, and Takeda. I. K. has received honoraria from AstraZeneca and educational/travel grants from Incyte, BeiGene, AstraZeneca, Janssen, and Pfizer. J. K. has received honoraria and/or travel/accommodation expenses from BMS, AbbVie, Sanofi, Pfizer, and Janssen. M. K. has received honoraria from GSK and Pfizer, and travel support from GSK, Janssen, Oncopeptides, Takeda, and Stemline. T. L. declares a consulting or advisory role for Janssen and has received research funding from Sanofi (to institution) and support for traveling, accommodations, and expenses from Sanofi, Lilly, Janssen, AOP Health, BeiGene, Alexion, and Regeneron. I. v. M. has participated in advisory boards and received honoraria from AbbVie, Janssen, BMS, GSK, Amgen, Sanofi, Pfizer, Oncopeptides, Stemline, and Takeda. C. S. M. declares a consulting or advisory role for BMS, Amgen, GSK, Janssen, and Sanofi, and has received honoraria and/or travel support from BMS, Amgen, GSK, Janssen, Sanofi, and Pfizer. S. T. has received honoraria from Janssen, Sanofi, Pfizer, Amgen, AbbVie, BMS, Menarini, Stemline, GSK, Takeda, and Kyowa Kirin. K. T. G. has received consulting fees from Sanofi, Takeda, Novartis, Amgen, GSK, and Janssen and honoraria from Novartis, Amgen, and GSK. R. W. consulted for and/or received honoraria from AbbVie, Alexion, Amgen, BMS, Janssen, Kite/Gilead, Novartis, Pfizer, Sanofi, and Takeda, and received research funding from Janssen and Sanofi (all paid to UKF). M. Hä. has received honoraria from SOBI, Novartis, Gilead, Falk Foundation, BMS, and Kite, and declares a consulting role for Pfizer, Incyte, Sanofi, Roche, Amgen, SOBI, Janssen, Kite, BMS, and BeiGene. L. R. declares a consulting or advisory role for BMS, Amgen, GSK, Janssen, Sanofi, and Pfizer, and has received research funding from BMS, travel support from Janssen and BeiGene, and honoraria from BMS, Janssen, Pfizer, and Sanofi. M. S. R. declares a consulting or advisory role for BMS, Amgen, GSK, Janssen, Sanofi, Pfizer, AbbVie, and Takeda, and has received research funding from BMS, Janssen, Sanofi, and Heidelberg Pharma; travel support from BMS, Amgen, and Janssen; and honoraria from BMS, Janssen, AbbVie, and Sanofi. R. T., J. B., F. Br., M. D., R. F., D. N. G., S. G. M., C. H., M. Hö., C. K., K. K., V. L., M. M., C. M. T., and R. Z. declare no potential competing interests.

## ETHICS STATEMENT

Each center obtained independent approval by the respective institutional committee or board and written informed consent according to local requirements. The vote of the Ethics Committee of the Medical Faculty of Heidelberg University is provided under S‐096/2017. All procedures in this study were conducted in accordance with the Declaration of Helsinki.

## FUNDING

J. H. F. is funded through the IMS and Paula and Rodger Riney Foundation. F. Ba. was supported by the German Research Foundation (DFG) under TRR 387/1 (project ID: 514894665), DFG BA 2851/6‐1 (project ID: 452409123), and DFG BA 2851/7‐1 (project ID: 537477296). M. D. was supported by the Deutsche Forschungsgemeinschaft (DFG) in the framework of the DFG Clinician Scientist Program (UMEA, FU 356/12‐2). S. T. received research support from a grant from the Bruno and Helene Jöster Foundation and the Thomas Kirch Foundation. This work was also in part supported by the Deutsche Forschungsgemeinschaft (German Research Consortium, DFG): No. SFB‐TRR 388/1 2021–452881907 (to S. T.) and No. 391587558 (to S. T.). We further acknowledge structural support from the Bavarian Cancer Research Center (BZKF) and the German Consortium for Translational Cancer Research (DKTK). L. R. is funded by the Rodger Riney Foundation and German Cancer Aid via the MSNZ program. M. S. R. has received research funding from the Dietmar Hopp Foundation.

## Supporting information

Supporting information.

## Data Availability

The datasets generated and analyzed during the current study are available from the corresponding author upon reasonable request.
