# Supplementary material for: A German multicenter real‐world analysis of talquetamab in 138 patients with relapsed/refractory multiple myeloma
Source: Hemasphere. 2025 Apr 17;9(4):e70114. doi: 10.1002/hem3.70114 (PMC12005056; doi:10.1002/hem3.70114)
Supplement: Supplementary file 1 — Supporting information. [file HEM3-9-e70114-s001.pdf]

Supplementary Materials for

A German multicenter real-world analysis of talquetamab in 138 patients with relapsed/refractory multiple myeloma

Supplementary Materials

- **Supplementary Figures S1-S2**
- **Supplementary Tables S1-S12**

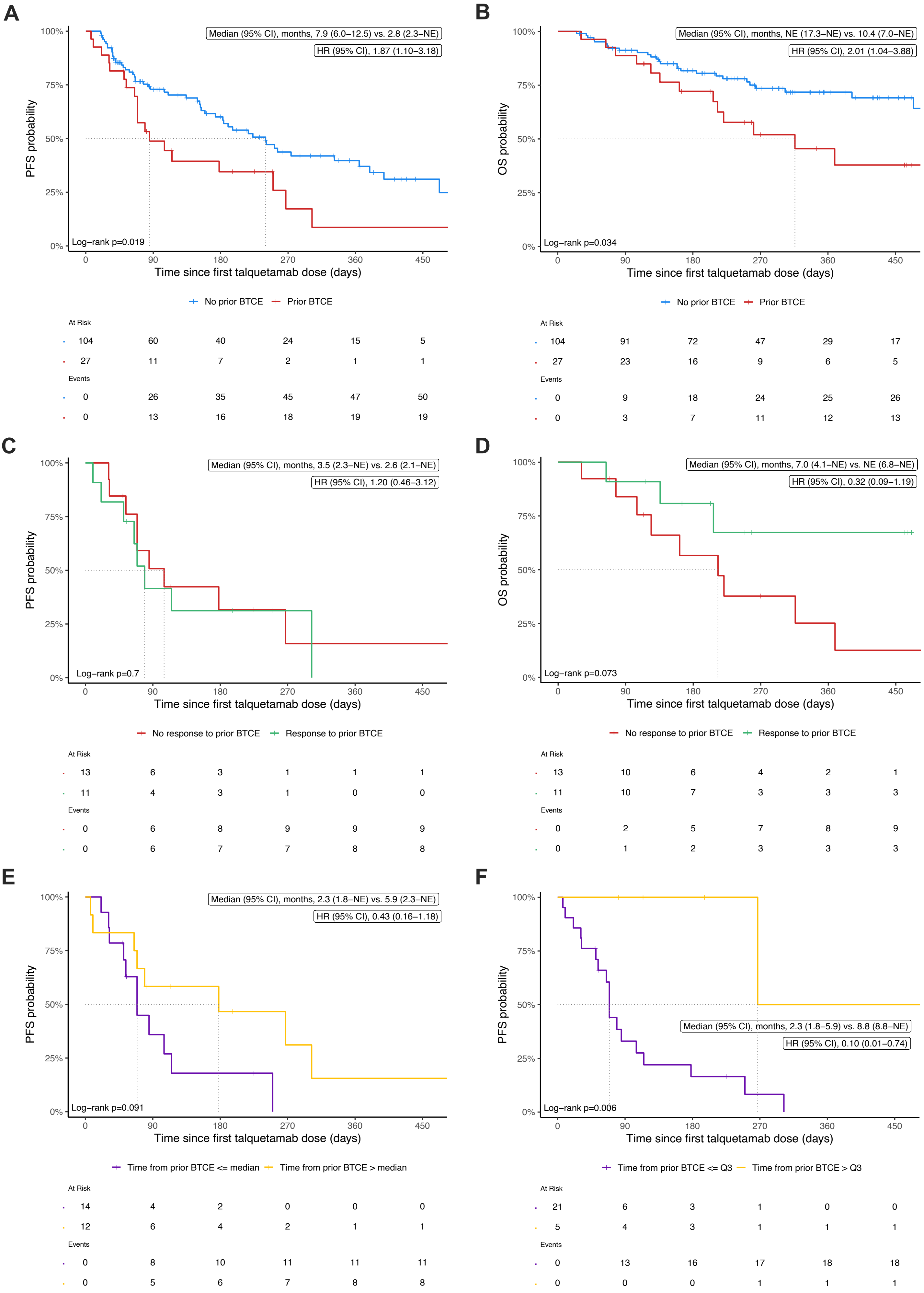

A
Median (95% CI), months, 7.9 (6.0–12.5) vs. 2.8 (2.3–NE)
HR (95% CI), 1.87 (1.10–3.18)
PFS probability
Log–rank p=0.019
Time since first talquetamab dose (days)
No prior BTCE
Prior BTCE
At Risk
104 60 40 24 15 5
27 11 7 2 1 1
Events
0 26 35 45 47 50
0 13 16 18 19 19
B
Median (95% CI), months, NE (17.3–NE) vs. 10.4 (7.0–NE)
HR (95% CI), 2.01 (1.04–3.88)
OS probability
Log–rank p=0.034
Time since first talquetamab dose (days)
No prior BTCE
Prior BTCE
At Risk
104 91 72 47 29 17
27 23 16 9 6 5
Events
0 9 18 24 25 26
0 3 7 11 12 13
C
Median (95% CI), months, 3.5 (2.3–NE) vs. 2.6 (2.1–NE)
HR (95% CI), 1.20 (0.46–3.12)
PFS probability
Log–rank p=0.7
Time since first talquetamab dose (days)
No response to prior BTCE
Response to prior BTCE
At Risk
13 6 3 1 1 1
11 4 3 1 0 0
Events
0 6 8 9 9 9
0 6 7 7 8 8
D
Median (95% CI), months, 7.0 (4.1–NE) vs. NE (6.8–NE)
HR (95% CI), 0.32 (0.09–1.19)
OS probability
Log–rank p=0.073
Time since first talquetamab dose (days)
No response to prior BTCE
Response to prior BTCE
At Risk
13 10 6 4 2 1
11 10 7 3 3 3
Events
0 2 5 7 8 9
0 1 2 3 3 3
E
Median (95% CI), months, 2.3 (1.8–NE) vs. 5.9 (2.3–NE)
HR (95% CI), 0.43 (0.16–1.18)
PFS probability
Log–rank p=0.091
Time since first talquetamab dose (days)
Time from prior BTCE <= median
Time from prior BTCE > median
At Risk
14 4 2 0 0 0
12 6 4 2 1 1
Events
0 8 10 11 11 11
0 5 6 7 8 8
F
Median (95% CI), months, 2.3 (1.8–5.9) vs. 8.8 (8.8–NE)
HR (95% CI), 0.10 (0.01–0.74)
PFS probability
Log–rank p=0.006
Time since first talquetamab dose (days)
Time from prior BTCE <= Q3
Time from prior BTCE > Q3
At Risk
21 6 3 1 0 0
5 4 3 1 1 1
Events
0 13 16 17 18 18
0 0 0 1 1 1

Supp. Figure S1 │ Kaplan-Meier estimates of progression-free survival (PFS) and overall survival (OS) for patients with prior bispecific T-cell engager (BTCE) therapy and corresponding subgroups.

A. Kaplan-Meier estimates of the probability of PFS for patients with no prior BTCE therapy (blue) and patients with prior BTCE therapy (red) (3-months PFS 73% vs. 49%; 6-months PFS 60% vs. 35%). **B.** Kaplan-Meier estimates of the probability of OS for patients with no prior BTCE therapy (blue) and patients with prior BTCE therapy (red) (3-months OS 91% vs. 89%; 6-months OS 82% vs. 72%). **C.** Kaplan-Meier estimates of the probability of PFS for patients with no response to prior BTCE therapy (red) and patients with response to prior BTCE therapy (green) (3-months PFS 51% vs. 42%; 6-months PFS 32% vs. 31%). Treatment response was defined as partial response or better. Patients whose response was not available or not evaluable were excluded. **D.** Kaplan-Meier estimates of the probability of OS for patients with no response to prior BTCE therapy (red) and patients with response to prior BTCE therapy (green) (3-months OS 84% vs. 91%; 6-months OS 57% vs. 81%). Treatment response was defined as partial response or better. Patients whose response was not available or not evaluable were excluded. **E.** Kaplan-Meier estimates of the probability of PFS for patients with prior BTCE therapy and time from last BTCE application to first talquetamab dose ≤ median (purple) and patients with prior BTCE therapy and time from last BTCE application to first talquetamab dose > median (yellow) (3-months PFS 36% vs. 58%; 6-months PFS 18% vs. 47%). Cut-off (median): 57 days (1.9 months). **F.** Kaplan-Meier estimates of the probability of PFS for patients with prior BTCE therapy and time from last BTCE application to first talquetamab dose ≤ upper/third quartile (Q_3) (purple) and patients with prior BTCE therapy and time from last BTCE application to first talquetamab dose > Q_3 (yellow) (3-months PFS 33% vs. 100%; 6-months PFS 17% vs. 100%). Cut-off (Q_3): 178 days (5.9 months).

The median survival times in months and the 95% confidence intervals (CI), the results of the Cox regression analysis (hazard ratio [HR] and 95% CI) and log-rank test, the number of evaluable patients at risk and the number of events are provided above or below the curves. BTCE, bispecific T-cell engager. CI, confidence interval. HR, hazard ratio. NE, not estimable. OS, overall survival. PFS, progression-free survival. Q_3, upper/third quartile.

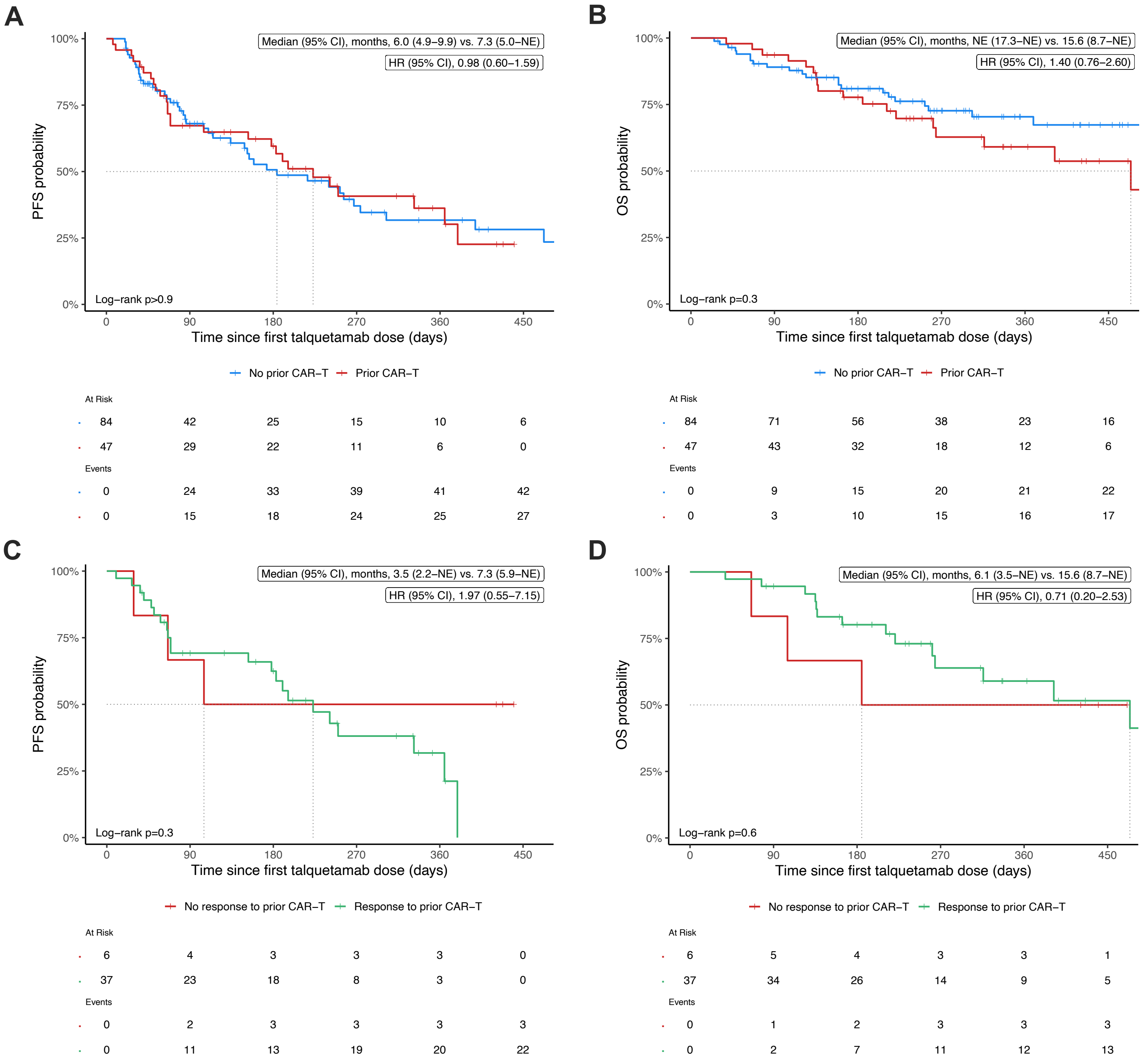

Supp. Figure S2 │ Kaplan-Meier estimates of progression-free survival (PFS) and overall survival (OS) for patients with prior chimeric antigen receptor (CAR) T-cell therapy and corresponding subgroups.

A. Kaplan-Meier estimates of the probability of PFS for patients with no prior CAR T-cell therapy (blue) and patients with prior CAR T-cell therapy (red) (3-months PFS 68% vs. 67%; 6-months PFS 51% vs. 60%). **B.** Kaplan-Meier estimates of the probability of OS for patients with no prior CAR T-cell therapy (blue) and patients with prior CAR T-cell therapy (red) (3-months OS 89% vs. 94%; 6-months OS 81% vs. 78%). **C.** Kaplan-Meier estimates of the probability of PFS for patients with no response to prior CAR T-cell therapy (red) and patients with response to prior CAR T-cell therapy (green) (3-months PFS 67% vs. 69%; 6-months PFS 50% vs. 62%). Treatment response was defined as partial response or better. Patients whose response was not available or not evaluable were excluded. **D.** Kaplan-Meier estimates of the probability of OS for patients with no response to prior CAR T-cell therapy (red) and patients with response to prior CAR T-cell therapy (green)

(3-months OS 83% vs. 95%; 6-months OS 67% vs. 80%). Treatment response was defined as partial response or better. Patients whose response was not available or not evaluable were excluded.

The median survival times in months and the 95% confidence intervals (CI), the results of the Cox regression analysis (hazard ratio [HR] and 95% CI) and log-rank test, the number of evaluable patients at risk and the number of events are provided above or below the curves. CAR, chimeric antigen receptor. CI, confidence interval. HR, hazard ratio. NE, not estimable. OS, overall survival. PFS, progression-free survival.

Supp. Table S1 │ Treatment application and status

	Real-world cohort<br>n = 138
**Indication**, no./total no. (%)	
Definitive / Long-term therapy	105/138 (76)
Bridging therapy to CAR T-cell therapy	32/138 (23)
Started before T-cell apheresis	18/138 (13)
Successful manufacturing	8/18 (44)
Out-of-specification product	3/18 (17)
Data on manufacturing not available (yet)	7/18 (39)
Started after T-cell apheresis	14/138 (10)
Successful manufacturing	11/14 (79)
Out-of-specification product	0/14 (0)
Manufacturing failure (at first attempt)	1/14 (7)
Data on manufacturing not available (yet)	2/14 (14)
Bridging therapy to another treatment and (then) to CAR T-cell therapy	1/138 (1)
**Administered doses**[a], no./total no. (%)	
Only step-up dose(s)	7/138 (5)
800 µg/kg Q2W as intended regimen	7/7 (100)
1 full dose[b]	12/138 (9)
800 µg/kg Q2W as intended regimen	12/12 (100)
> 1 full dose[c]	119/138 (86)
400 µg/kg QW as regimen at the start of treatment and/or on the last day of treatment	6/119 (5)
800 µg/kg Q2W as regimen at the start of treatment and/or on the last day of treatment	104/119 (87)
regimen not specified	6/119 (5)
**Primary reason why patients had only received step-up doses or only 1 full dose**, no./total no. (%)	
Death (any reason)	5/19 (26)
Progressive disease	4/19 (21)
Severe CRS/ICANS	2/19 (11)
Other adverse events	7/19 (37)
Other reason	1/19 (5)
**Treatment intervals among patients with > 1 full dose**, no./total no. (%)	
Every week	4/119 (3)
Every 2 weeks	79/119 (66)
Switched to every 3 weeks	5/119 (4)
Switched to every ≥ 4 weeks	31/119 (26)
switched between C1-3	11/119 (9)
switched between C4-6	12/119 (10)
switched after C6	8/119 (7)
**Primary reason for switching of treatment intervals**, no./total no. (%)	
Side effects	29/36 (81)
Patient preference	3/36 (8)
Adequate response	2/36 (6)
Reduction of (possible) T cell exhaustion	1/36 (3)
Not specified	1/36 (3)
**Reduction of the absolute treatment dose**[d], no./total no. (%)	
No	116/132 (88)
Yes	16/132 (12)

Unknown	6
**Reason(s) for dose reduction**[e], no./total no. (%)	
Skin-/Nail-related toxicity	7/16 (44)
Dysgeusia	5/16 (31)
Mucositis	2/16 (13)
Fever/Infection	4/16 (25)
Other adverse events	4/16 (25)
Long treatment break	2/16 (13)
Other reason	1/16 (6)
**Treatment break > 28 days**, no./total no. (%)	
No	111/137 (81)
Yes	26/137 (19)
Unknown	1[f]
**Reason(s) for treatment break > 28 days**[e], no./total no. (%)	
Infection	12/26 (46)
Skin-/Nail-related toxicity	7/26 (27)
Dysgeusia	6/26 (23)
Other adverse events	8/26 (31)
Other reason	1/26 (4)
**Outcome of the adverse event(s) related to treatment break**, no./total no. (%)	
Fatal (death)	1/25 (4)
Not recovered or not resolved	2/25 (8)
Recovered or resolved	17/25 (68)
Recovered or resolved with sequelae	4/25 (16)
Recovering or resolving	0/25 (0)
Not specified	1/25 (4)
**Second step-up dosing**, no./total no. (%)	
No	128/138 (93)
Yes	10[g]/138 (7)
**Treatment discontinuation,** no./total no. (%)	
No	35/137 (26)
Yes	102/137 (74)
Unknown	1[h]
**Reason(s) for treatment discontinuation**[e], no./total no. (%)	
Progressive disease, change to BSC concept or death	67/102 (66)
Only used as bridging therapy	17/102 (17)
Infection	3/102 (3)
Other adverse events	12/102 (12)
Second malignancy	4/102 (4)
Other reason	1/102 (1)

a. The following treatment regimens are approved in Germany: 1. 400 μg/kg administered subcutaneously every week (QW), after step-up dosing with 10, 60 and 400 μg/kg (first full dose). 2. 800 μg/kg administered subcutaneously every two weeks (Q2W), after step-up dosing with 10, 60, 400 and 800 μg/kg (first full dose). b. n=3 patients received one full dose of the intended regimen and continued treatment with one reduced dose (n=2) or two reduced doses (n=1) (each with two-week intervals). c. The following regimens were used at the start of treatment: 400 μg/kg QW (n=6), 400 μg/kg Q2W (n=2), 800 μg/kg Q2W (n=102), others (n=3) or not specified (n=6). The following regimens were used on the last day of treatment: 400 μg/kg QW (n=4), 400 μg/kg Q2W (n=5), 400 μg/kg Q3W (n=1), 400 μg/kg Q4W (n=2), 800 μg/kg Q2W (n=73), 800 μg/kg Q3W (n=2), 800 μg/kg Q4W (n=24), 800 μg/kg Q5W (n=1), others (n=1) or not specified (n=6). d. Repetition of doses during the initial step-up dosing (n=4) or a repeated/second step-up dosing period were cot counted as dose reductions. Data for the latter constellation are reported separately. e. Multiple answers possible. f. Lost to follow-up (external continuation of talquetamab treatment with limited data access). g. There were no cases of CRS/ICANS among the patients with available data (n=8; missing data on complications: n=2). h. Lost to follow-up (further care provided externally, unclear treatment status). BSC, best supportive care. C, cycle. CAR, chimeric antigen receptor. CRS, cytokine release syndrome. ICANS, immune effector cell-associated neurotoxicity syndrome.

Supp. Table S2 │ Baseline laboratory parameters and cytopenias (prior to first talquetamab dose)

	**Real-world cohort n = 138**
**Baseline laboratory parameters**[a], median (IQR)	
B2-MG, mg/l	4.5 (3.1-7.5), n = 109
LDH, U/l	250 (196-330)
LDH ≤ ULN, no./total no. (%)	73/138 (53)
LDH > ULN, no./total no. (%)	65/138 (47)
Creatinine, mg/dl	1.10 (0.90-1.40)
eGFR, ml/min	61 (48-84), n = 135
≥ 60, no./total no. (%)	73/138 (53)
30-59, no./total no. (%)	52/138 (38)
< 30, no./total no. (%)	13/138 (9)
Hemoglobin, g/dl	9.95 (8.60-11.70)
Platelet count, cells/nl	112 (56-193)
Absolute neutrophil count, cells/nl	2.56 (1.61-3.73), n = 136
Absolute lymphocyte count, cells/nl	0.96 (0.50-1.36), n = 136
**Baseline cytopenias**[a], no./total no. (%)	
Anemia	
None	14/138 (10)
Any	124/138 (90)
Grade 1	55/138 (40)
Grade 2	50/138 (36)
Grade ≥ 3	19/138 (14)
Thrombocytopenia	
None	54/138 (39)
Any	84/138 (61)
Grade 1	32/138 (23)
Grade 2	25/138 (18)
Grade 3	12/138 (9)
Grade 4	15/138 (11)
Neutropenia	
None	98/136 (72)
Any	38/136 (28)
Grade 1	8/136 (6)
Grade 2	17/136 (13)
Grade 3	8/136 (6)
Grade 4	5/136 (4)
Lymphocytopenia	
None	64/136 (47)
Any	72/136 (53)
Grade 1	15/136 (11)
Grade 2	24/136 (18)
Grade 3	23/136 (17)
Grade 4	10/136 (7)

a. Determined prior to first dose of talquetamab. B2-MG, beta-2-microglobulin. eGFR, estimated glomerular filtration rate (CKD-EPI). IQR, interquartile range. LDH, lactate dehydrogenase. ULN, upper limit of normal.

Supp. Table S3 │ MonumenTAL-1 eligibility criteria

	Real-world cohort n = 138
**Fulfillment of eligibility criteria**[a], no./total no. (%)	
Yes	56/134 (42)
No	78/134 (58)
Did not meet 1 criterion	41/134 (31)
Did not meet ≥ 2 criteria	34/134 (25)
Not specified	3/134 (2)
**Reasons for non-eligiblity**[b], no./total no. (%)	
Not measurable	4/134 (3)
ECOG ≥ 2	9/134 (7)
Anemia[c]	17/134 (13)
Thrombocytopenia[d]	30/134 (22)
Neutropenia[e]	4/134 (3)
Renal dysfunction[f]	20/134 (15)
Hepatic dysfunction[g]	0/134 (0)
Recent allogeneic SCT, immunosuppressive drugs or GVHD[h]	3/134 (2)
Recent autologous SCT[i]	2/134 (1)
Glucocorticoid treatment[j]	8/134 (6)
Other prohibited prior antitumor therapies[k]	18/134 (13)
CNS or meningeal involvement	3/134 (2)
PCL or amyloidosis	2/134 (1)
HIV or hepatitis B	2/134 (1)
Other serious medical condition	3/134 (2)
Not specified	3/134 (2)

a. At the day of informed consent. Criteria were adapted from the original study protocol published by Chari et al., 2022. b. Multiple answers possible. c. Hemoglobin < 8 g/dl or red blood cell transfusion within 7 days prior to laboratory test. d. Platelets < 75/nl or platelet transfusion within 7 days prior to laboratory test. e. Neutrophils < 1/nl or growth factor support prior to laboratory test. f. Creatinine clearance < 40 ml/min/1.73 m^2 according to MDRD formula. g. Aspartate or alanine aminotransferase > 3 x upper limit of normal. h. Modified criterion based on later amendment: allogeneic SCT within 6 months before first dose of talquetamab and no immunosuppressive drugs for 6 weeks without GVHD signs. i. ≤ 12 weeks before first dose of talquetamab. j. Cumulative dose of glucocorticoids equivalent to ≥ 140 mg of prednisone within 14 days before first dose of talquetamab. k. Including targeted/epigenetic/investigational therapies (within 21 days or 5 half-lives before first dose of talquetamab), monoclonal antibiodies (within 21 days), cytotoxic agents (within 21 days), proteasome inhibitors (within 14 days), immunomodulatory agents (within 7 days) or radiotherapy (within 21 days and with relevance for bone marrow reserve). CNS, central nervous system. ECOG, Eastern Cooperative Oncology Group performance status. GVHD, graft-versus-host disease. HIV, human immunodeficiency virus. PCL, plasma cell leukemia. SCT, stem cell transplant.

Supp. Table S4 │ Univariate logistic regression analysis of grade ≥ 2 cytokine release syndrome (CRS)

Characteristic	N	Event N	OR	95% CI	p value
**Eligibility**					
No	78	24	—	—	
Yes	55	7	0,33	0.12, 0.79	**0,019**
**Age [cont.]**	137	31	0,99	0.95, 1.03	0,68
**Age ≥ 70 years**					
No	100	25	—	—	
Yes	37	6	0,58	0.20, 1.48	0,28
**Gender**					
Male	95	18	—	—	
Female	42	13	1,92	0.82, 4.40	0,12
**ECOG ≥ 2**[a]					
No	100	17	—	—	
Yes	32	13	3,34	1.38, 8.08	**0,007**
**Extramedullary disease**[a]					
No	64	13	—	—	
Yes	59	17	1,59	0.70, 3.70	0,27
**Extraosseous disease**[a,b]					
No	78	16	—	—	
Yes	45	14	1,75	0.75, 4.06	0,19
**Bone marrow burden ≥ 60%**[a]					
No	34	9	—	—	
Yes	15	1	0,2	0.01, 1.22	0,14
**High risk cytogenetics**[c]					
No	62	9	—	—	
Yes	57	19	2,94	1.23, 7.50	**0,018**
**+1q**[c,d]					
No	54	12	—	—	
Yes	58	14	1,11	0.46, 2.72	0,81
**ISS stage III**[a]					
No	65	9	—	—	
Yes	48	15	2,83	1.13, 7.42	**0,029**
**R-ISS stage III**[a]					
No	75	12	—	—	
Yes	34	12	2,86	1.12, 7.39	**0,028**
**Increased LDH (> ULN)**[a]					
No	73	13	—	—	
Yes	64	18	1,81	0.81, 4.13	0,15
**eGFR < 30 ml/min**[a]					
No	124	26	—	—	
Yes	13	5	2,36	0.66, 7.68	0,16
**Prior therapy lines > median (> 6)**					
No	91	22	—	—	
Yes	46	9	0,76	0.31, 1.78	0,54

**Prior allogeneic SCT**					
No	121	27	—	—	
Yes	16	4	1,16	0.31, 3.64	0,81
**Triple-class refractoriness**[e]					
No	20	4	—	—	
Yes	117	27	1,2	0.40, 4.46	0,76
**Penta-drug refractoriness**[f]					
No	72	18	—	—	
Yes	63	13	0,78	0.34, 1.75	0,55
**Prior BCMA-targeted therapy**[g]					
No	67	15	—	—	
Yes	70	16	1,03	0.46, 2.30	0,95
**Prior BTCE therapy**					
No	110	24	—	—	
Yes	27	7	1,25	0.45, 3.21	0,65
**Time (days) from last BTCE application to first talquetamab dose [cont.]**	26	6	0,99	0.98, 1.00	0,35
**Prior CAR T-cell therapy**					
No	90	21	—	—	
Yes	47	10	0,89	0.37, 2.05	0,78
**Time (days) from last CAR-T infusion to first talquetamab dose [cont.]**	46	10	1	1.0, 1.00	0,48
**Prior T-cell redirecting immunotherapy**[h]					
No	72	18	—	—	
Yes	65	13	0,75	0.33, 1.67	0,49
**Classical cytotoxic chemotherapy ≤ 60 days prior to first talquetamab dose**					
No	102	25	—	—	
Yes	33	6	0,68	0.23, 1.76	0,45
**Classical cytotoxic polychemotherapy**[i] **≤ 60 days prior to first talquetamab dose**					
No	122	29	—	—	
Yes	15	2	0,49	0.07, 1.93	0,37
**Body mass index [cont.]**[a]	135	31	0,95	0.87, 1.04	0,29
**Overweight**[a,j]					
No	67	18	—	—	
Yes	68	13	0,64	0.28, 1.44	0,29

a. Determined prior to first dose of talquetamab. b. Extraosseous disease was defined as the presence of bone-independent/organ-infiltrating disease manifestations or plasma cell leukemia (+/- other extramedullary disease manifestations). Only bone-associated (paramedullary) disease manifestations were not classified as extraosseous disease. c. Based on the latest available cytogenetic findings. d. Chromosome 1q gain (3 copies) or amplification (≥ 4 copies). e. Refractory to at least one immunomodulatory agent, at least one proteasome inhibitor and at least one anti-CD38 monoclonal antibody. f. Refractory to at least two proteasome inhibitors, at least two immunomodulatory agents and at least one anti-CD38 monoclonal antibody. g. Including antibody-drug conjugates, bispecific antibodies and CAR T-cell therapy. h. Including bispecific antibodies and CAR T-cell therapy. i. Combination of ≥ 2 classical cytotoxic agents. j. Body mass index ≥ 25. BCMA, B-cell maturation antigen. BTCE, bispecific T-cell engager. CAR, chimeric antigen receptor. CI, confidence interval. ECOG, Eastern Cooperative Oncology Group performance status. eGFR, estimated glomerular filtration rate (CKD-EPI). ISS, International Staging System. LDH, lactate dehydrogenase. OR, odds ratio. R-ISS, Revised International Staging System. SCT, stem cell transplant. ULN, upper limit of normal.

Supp. Table S5 │ Multivariate logistic regression analysis of grade ≥ 2 cytokine release syndrome (CRS)

	Grade ≥ 2 cytokine release syndrome (CRS)		
**Variable**	**OR**	**95% CI**	**p value**
Age [cont.]	0.99	0.94-1.04	0.63
ECOG ≥ 2[a]	3.63	1.05-12.9	**0.041**
Extramedullary disease[a]	2.19	0.69-7.44	0.19
High risk cytogenetics[b]	4.49	1.38-16.7	**0.016**
ISS stage III[a]	2.55	0.82-8.39	0.11
Increased LDH (> ULN)[a]	1.02	0.31-3.26	0.97

a. Determined prior to first dose of talquetamab. b. based on the latest available cytogenetic findings. CI, confidence interval. ECOG, Eastern Cooperative Oncology Group performance status. ISS, International Staging System. LDH, lactate dehydrogenase. OR, odds ratio. ULN, upper limit of normal.

Supp. Table S6 │ Univariate logistic regression analysis of any grade neurotoxicity (ICANS)

Characteristic	N	Event N	OR	95% CI	p value
**Eligibility**					
No	77	8	—	—	
Yes	56	4	0,66	0.17, 2.23	0,52
**Age [cont.]**	137	12	1,04	0.98, 1.12	0,21
**Age ≥ 70 years**					
No	100	7	—	—	
Yes	37	5	2,08	0.58, 6.97	0,24
**Gender**					
Male	96	7	—	—	
Female	41	5	1,77	0.49, 5.90	0,36
**ECOG ≥ 2**[a]					
No	100	5	—	—	
Yes	32	6	4,38	1.23, 16.3	**0,022**
**Extramedullary disease**[a]					
No	64	6	—	—	
Yes	59	6	1,09	0.32, 3.70	0,88
**Extraosseous disease**[a,b]					
No	78	6	—	—	
Yes	45	6	1,85	0.54, 6.28	0,32
**Bone marrow burden ≥ 60%**[a]					
No	34	5	—	—	
Yes	15	1	0,41	0.02, 2.90	0,44
**High risk cytogenetics**[c]					
No	63	6	—	—	
Yes	56	5	0,93	0.25, 3.27	0,91
**+1q**[c,d]					
No	53	5	—	—	
Yes	59	6	1,09	0.31, 3.99	0,9
**ISS stage III**[a]					
No	65	1	—	—	
Yes	48	9	14,8	2.63, 278	**0,012**
**R-ISS stage III**[a]					
No	75	5	—	—	
Yes	34	4	1,87	0.44, 7.53	0,38
**Increased LDH (> ULN)**[a]					
No	73	7	—	—	
Yes	64	5	0,8	0.23, 2.64	0,71
**eGFR < 30 ml/min**[a]					
No	124	12	—	—	
Yes	13	0			
**Prior therapy lines > median (> 6)**					
No	90	10	—	—	
Yes	47	2	0,36	0.05, 1.42	0,19

**Prior allogeneic SCT**					
No	122	12	—	—	
Yes	15	0			
**Triple-class refractoriness**[e]					
No	20	2	—	—	
Yes	117	10	0,84	0.20, 5.76	0,83
**Penta-drug refractoriness**[f]					
No	71	8	—	—	
Yes	64	4	0,53	0.13, 1.76	0,31
**Prior BCMA-targeted therapy**[g]					
No	66	9	—	—	
Yes	71	3	0,28	0.06, 0.99	0,065
**Prior BTCE therapy**					
No	109	11	—	—	
Yes	28	1	0,33	0.02, 1.81	0,3
**Time (days) from last BTCE application to first talquetamab dose [cont.]**	27	1			
**Prior CAR T-cell therapy**					
No	90	10	—	—	
Yes	47	2	0,36	0.05, 1.42	0,19
**Time (days) from last CAR-T infusion to first talquetamab dose [cont.]**	46	2			
**Prior T-cell redirecting immunotherapy**[h]					
No	71	9	—	—	
Yes	66	3	0,33	0.07, 1.16	0,11
**Classical cytotoxic chemotherapy ≤ 60 days prior to first talquetamab dose**					
No	102	11	—	—	
Yes	33	1	0,26	0.01, 1.41	0,2
**Classical cytotoxic polychemotherapy**[i] **≤ 60 days prior to first talquetamab dose**					
No	122	11	—	—	
Yes	15	1	0,72	0.04, 4.15	0,76
**CRS (any grade)**					
No	41	2	—	—	
Yes	95	9	2,04	0.50, 13.8	0,38
**CRS grade ≥ 2**					
No	106	5	—	—	
Yes	30	6	5,05	1.41, 18.9	**0,012**
**Body mass index [cont.]**[a]	135	12	1,02	0.90, 1.14	0,7
**Overweight**[a,j]					
No	66	7	—	—	
Yes	69	5	0,66	0.19, 2.17	0,5

a. Determined prior to first dose of talquetamab. b. Extraosseous disease was defined as the presence of bone-independent/organ-infiltrating disease manifestations or plasma cell leukemia (+/- other extramedullary disease manifestations). Only bone-associated (paramedullary) disease manifestations were not classified as extraosseous disease. c. Based on the latest available cytogenetic findings. d. Chromosome 1q gain (3 copies) or amplification (≥ 4 copies). e. Refractory to at least one immunomodulatory agent, at least one proteasome inhibitor and at least one anti-CD38 monoclonal antibody. f. Refractory to at least two proteasome inhibitors, at least two immunomodulatory agents and at least one anti-CD38 monoclonal antibody. g. Including antibody-drug conjugates, bispecific antibodies and CAR T-cell therapy. h. Including bispecific antibodies and CAR T-cell therapy. i. Combination of ≥ 2 classical cytotoxic agents. j. Body mass index ≥ 25. BCMA, B-cell maturation antigen. BTCE, bispecific T-cell engager. CAR, chimeric antigen receptor. CI, confidence interval. CRS, cytokine release syndrome. ECOG, Eastern Cooperative Oncology Group performance status. eGFR, estimated glomerular filtration rate (CKD-EPI). ISS, International Staging System. LDH, lactate dehydrogenase. OR, odds ratio. R-ISS, Revised International Staging System. SCT, stem cell transplant. ULN, upper limit of normal.

Supp. Table S7 | Opportunistic infections

	Real-world cohort<br>n = 138
**Opportunistic infection**, No. (%)	
None	104/131 (79)
Any	27/131 (21)
Before the first full dose	14/131 (11)
After the first full dose	13/131 (10)
**Pathogens**[a], No. (%)	
Viral opportunistic infections	15/131 (11)
CMV	7/131 (5)
EBV	2/131 (2)
VZV	2/131 (2)
RSV	3/131 (2)
Others	2/131 (2)
Fungal opportunistic infections	7/131 (5)
Candida spp.	5/131 (4)
Others	2/131 (2)
Bacterial opportunistic infections	9/131 (7)

a. Multiple answers possible.

Supp. Table S8 | Efficacy

	Real-world cohort[a] n = 131	MonumenTAL-1 cohort[b] n = 44	P value
**Best overall response**, No. (%)			
≥ CR	3/123 (2.4)	10/44 (22.7)	
nCR	29/123 (23.6)		
VGPR	33/123 (26.8)	13/44 (29.5)	
PR	15/123 (12.2)	5/44 (11.4)	
MR	6/123 (4.9)		
SD	11/123 (8.9)	13/44 (29.5)	
PD	23/123 (18.7)	3/44 (6.8)	
Not available	3/123 (2.4)		
Not evaluable, no PD	8		
Treatment response (≥ PR)	80/123 (65.0)	28/44 (63.6)	0.86
≥ VGPR	65/123 (52.8)	23/44 (52.3)	> 0.99
**Time to first response**, months, median (range)	1.0 (0.2-5.7), n = 75	1.2 (0.3-6.8)	
**Time to best response**, months, median (range)	1.8 (0.4-12.1), n = 73		
**Duration of response**, months, median (95% CI)	12.7 (9.5-NE)	7.8 (4.6-NE)	
**Progression-free survival**, months, median (95% CI)	6.4 (5.1-9.0)		
**Overall survival**, months, median (95% CI)	NE (15.6-NE)		
**Follow-up time**, months, median (95% CI)	8.2 (6.9-9.4)[c]	4.2	

a. Only patients who had received ≥ 1 full dose were included in the efficacy analysis. b. Patients who had received subcutaneous talquetamab 800 μg/kg every two weeks (Chari et al., 2022). c. Median follow-up time for the total real-world cohort: 7.8 months (95% CI 6.8-8.7). CI, confidence interval. CR, complete response. MR, minimal response. nCR, near complete response. NE, not estimable. PD, progressive disease. PR, partial response. SD, stable disease. VGPR, very good partial response.

Supp. Table S9 │ Univariate logistic regression analysis of treatment response (≥ partial response)

Characteristic	N	Event N	OR	95% CI	p value
**Eligibility**					
No	67	41	—	—	
Yes	54	38	1,51	0.71, 3.27	0,29
**Age [cont.]**	123	80	1	0.97, 1.04	0,89
**Age ≥ 70 years**					
No	88	55	—	—	
Yes	35	25	1,5	0.65, 3.63	0,35
**Gender**					
Male	88	54	—	—	
Female	35	26	1,82	0.78, 4.53	0,18
**ECOG ≥ 2**[a]					
No	93	64	—	—	
Yes	26	13	0,45	0.19, 1.10	0,08
**Extramedullary disease**[a]					
No	61	45	—	—	
Yes	48	23	0,33	0.14, 0.72	**0,006**
**Extraosseous disease**[a,b]					
No	74	53	—	—	
Yes	35	15	0,3	0.13, 0.68	**0,005**
**Bone marrow burden ≥ 60%**[a]					
No	30	23	—	—	
Yes	15	8	0,35	0.09, 1.30	0,12
**High risk cytogenetics**[c]					
No	59	39	—	—	
Yes	47	31	0,99	0.44, 2.25	0,99
**+1q**[c,d]					
No	52	31	—	—	
Yes	50	35	1,58	0.70, 3.64	0,27
**ISS stage III**[a]					
No	57	45	—	—	
Yes	44	23	0,29	0.12, 0.69	**0,006**
**R-ISS stage III**[a]					
No	67	49	—	—	
Yes	31	16	0,39	0.16, 0.95	**0,039**
**Increased LDH (> ULN)**[a]					
No	65	48	—	—	
Yes	58	32	0,44	0.20, 0.92	**0,032**
**eGFR < 30 ml/min**[a]					
No	111	73	—	—	
Yes	12	7	0,73	0.22, 2.61	0,61
**Prior therapy lines > median (> 6)**					
No	78	55	—	—	
Yes	45	25	0,52	0.24, 1.12	0,1

**Prior allogeneic SCT**					
No	108	71	—	—	
Yes	15	9	0,78	0.26, 2.49	0,66
**Triple-class refractoriness[e]**					
No	17	14	—	—	
Yes	106	66	0,35	0.08, 1.17	0,12
**Penta-drug refractoriness[f]**					
No	61	49	—	—	
Yes	60	31	0,26	0.11, 0.58	**0,001**
**Prior BCMA-targeted therapy[g]**					
No	55	39	—	—	
Yes	68	41	0,62	0.29, 1.32	0,22
**Prior BTCE therapy**					
No	96	71	—	—	
Yes	27	9	0,18	0.07, 0.43	**<0.001**
**BTCE as last therapy line**					
No	10	4	—	—	
Yes	17	5	0,63	0.12, 3.34	0,57
**Response to prior BTCE**					
No	13	5	—	—	
Yes	11	3	0,6	0.10, 3.33	0,56
**Time (days) from last BTCE application to first talquetamab dose [cont.]**	26	8	1,01	1.00, 1.02	**0,05**
**Time from prior BTCE therapy to first talquetamab dose > median**					
No	14	3	—	—	
Yes	12	5	2,62	0.49, 16.4	0,27
**Time from prior BTCE therapy to first talquetamab dose > Q_3**					
No	21	4	—	—	
Yes	5	4	17	1.91, 388	**0,023**
**Prior CAR T-cell therapy**					
No	78	49	—	—	
Yes	45	31	1,31	0.61, 2.91	0,5
**Response to prior CAR T-cell therapy**					
No	6	4	—	—	
Yes	35	25	1,25	0.16, 7.54	0,81
**Time (days) from last CAR-T infusion to first talquetamab dose [cont.]**	45	31	1	1.00, 1.01	0,16
**Prior T-cell redirecting immunotherapy[h]**					
No	60	43	—	—	
Yes	63	37	0,56	0.26, 1.19	0,13
**Classical cytotoxic chemotherapy ≤ 60 days prior to first talquetamab dose**					
No	93	63	—	—	
Yes	28	15	0,55	0.23, 1.31	0,17
**Classical cytotoxic polychemotherapy[i] ≤ 60 days prior to first talquetamab dose**					
No	110	72	—	—	
Yes	13	8	0,84	0.26, 2.96	0,78

**CRS (any grade)**					
No	38	22	—	—	
Yes	85	58	1,56	0.70, 3.44	0,27
**CRS grade ≥ 2**					
No	97	63	—	—	
Yes	26	17	1,02	0.42, 2.62	0,97
**ICANS (any grade)**					
No	114	77	—	—	
Yes	8	3	0,29	0.06, 1.24	0,1
**Tocilizumab**					
No	81	52	—	—	
Yes	41	27	1,08	0.49, 2.41	0,86
**Dexamethasone**					
No	101	67	—	—	
Yes	21	12	0,68	0.26, 1.81	0,42
**Dysgeusia (any grade)**					
No	30	17	—	—	
Yes	91	63	1,72	0.73, 4.02	0,21
**Body mass index [cont.]**[a]	121	78	1,07	0.99, 1.17	0,11
**Overweight**[a,j]					
No	60	34	—	—	
Yes	61	44	1,98	0.93, 4.28	0,077

a. Determined prior to first dose of talquetamab. b. Extraosseous disease was defined as the presence of bone-independent/organ-infiltrating disease manifestations or plasma cell leukemia (+/- other extramedullary disease manifestations). Only bone-associated (paramedullary) disease manifestations were not classified as extraosseous disease. c. Based on the latest available cytogenetic findings. d. Chromosome 1q gain (3 copies) or amplification (≥ 4 copies). e. Refractory to at least one immunomodulatory agent, at least one proteasome inhibitor and at least one anti-CD38 monoclonal antibody. f. Refractory to at least two proteasome inhibitors, at least two immunomodulatory agents and at least one anti-CD38 monoclonal antibody. g. Including antibody-drug conjugates, bispecific antibodies and CAR T-cell therapy. h. Including bispecific antibodies and CAR T-cell therapy. i. Combination of ≥ 2 classical cytotoxic agents. j. Body mass index ≥ 25. BCMA, B-cell maturation antigen. BTCE, bispecific T-cell engager. CAR, chimeric antigen receptor. CI, confidence interval. CRS, cytokine release syndrome. ECOG, Eastern Cooperative Oncology Group performance status. eGFR, estimated glomerular filtration rate (CKD-EPI). ICANS, immune effector cell-associated neurotoxicity. ISS, International Staging System. LDH, lactate dehydrogenase. OR, odds ratio. R-ISS, Revised International Staging System. SCT, stem cell transplant. ULN, upper limit of normal. Q_3, upper/third quartile.

Supp. Table S10 │ Univariate Cox regression analysis of progression-free survival

Characteristic	N	Event N	HR	95% CI	p value
**Eligibility**					
No	73	46	—	—	
Yes	54	23	0,61	0.37, 1.00	0,05
**Age [cont.]**	131	70	1	0.97, 1.02	0,75
**Age ≥ 70 years**					
No	96	53	—	—	
Yes	35	17	0,71	0.41, 1.23	0,22
**Gender**					
Male	92	48	—	—	
Female	39	22	0,89	0.54, 1.48	0,66
**ECOG ≥ 2**[a]					
No	100	49	—	—	
Yes	27	19	1,72	1.01, 2.92	**0,046**
**Extramedullary disease**[a]					
No	63	26	—	—	
Yes	54	38	2,72	1.60, 4.62	**<0.001**
**Extraosseous disease**[a,b]					
No	77	31	—	—	
Yes	40	33	3,71	2.18, 6.30	**<0.001**
**Bone marrow burden ≥ 60%**[a]					
No	31	16	—	—	
Yes	15	8	1,74	0.73, 4.14	0,21
**High risk cytogenetics**[c]					
No	60	31	—	—	
Yes	53	28	1,07	0.64, 1.79	0,79
**+1q**[c,d]					
No	53	30	—	—	
Yes	53	28	0,93	0.55, 1.56	0,78
**ISS stage III**[a]					
No	64	26	—	—	
Yes	44	26	1,7	0.98, 2.94	0,057
**R-ISS stage III**[a]					
No	73	33	—	—	
Yes	31	18	1,41	0.79, 2.52	0,25
**Increased LDH (> ULN)**[a]					
No	71	31	—	—	
Yes	60	39	2,17	1.35, 3.49	**0,001**
**eGFR < 30 ml/min**[a]					
No	119	62	—	—	
Yes	12	8	1,27	0.61, 2.65	0,53

**Prior therapy lines > median (> 6)**					
No	85	38	—	—	
Yes	46	32	1,75	1.09, 2.80	**0,021**
**Prior allogeneic SCT**					
No	115	60	—	—	
Yes	16	10	1,03	0.52, 2.01	0,94
**Triple-class refractoriness**[e]					
No	19	8	—	—	
Yes	112	62	2,07	0.98, 4.35	0,056
**Penta-drug refractoriness**[f]					
No	68	30	—	—	
Yes	61	38	1,82	1.13, 2.94	**0,015**
**Prior BCMA-targeted therapy**[g]					
No	61	27	—	—	
Yes	70	43	1,22	0.76, 1.98	0,41
**Prior BTCE therapy**					
No	104	51	—	—	
Yes	27	19	1,87	1.10, 3.18	**0,021**
**BTCE as last therapy line**					
No	10	7	—	—	
Yes	17	12	1,17	0.46, 3.01	0,74
**Response to prior BTCE**					
No	13	9	—	—	
Yes	11	8	1,2	0.46, 3.12	0,71
**Time (days) from last BTCE application to first talquetamab dose [cont.]**	26	19	1	0.99, 1.00	0,061
**Time from prior BTCE therapy to first talquetamab dose > median**					
No	14	11	—	—	
Yes	12	8	0,43	0.16, 1.18	0,1
**Time from prior BTCE therapy to first talquetamab dose > Q_3**					
No	21	18	—	—	
Yes	5	1	0,1	0.01, 0.74	**0,025**
**Prior CAR T-cell therapy**					
No	84	43	—	—	
Yes	47	27	0,98	0.60, 1.59	0,93
**Response to prior CAR T-cell therapy**					
No	6	3	—	—	
Yes	37	22	1,97	0.55, 7.15	0,3
**Time (days) from last CAR-T infusion to first talquetamab dose [cont.]**	46	27	1	1.00, 1.00	0,38
**Prior T-cell redirecting immunotherapy**[h]					
No	66	32	—	—	
Yes	65	38	1,15	0.72, 1.85	0,56

**Classical cytotoxic chemotherapy ≤ 60 days prior to first talquetamab dose**					
No	97	47	—	—	
Yes	32	22	1,84	1.10, 3.06	**0,019**
**Classical cytotoxic polychemotherapy[i] ≤ 60 days prior to first talquetamab dose**					
No	116	58	—	—	
Yes	15	12	1,68	0.90, 3.14	0,1
**CRS (any grade)**					
No	41	23	—	—	
Yes	90	47	0,91	0.55, 1.50	0,72
**CRS grade ≥ 2**					
No	104	53	—	—	
Yes	27	17	1,11	0.64, 1.92	0,72
**ICANS (any grade)**					
No	122	62	—	—	
Yes	8	7	2,99	1.36, 6.61	**0,007**
**Tocilizumab**					
No	87	46	—	—	
Yes	43	24	1,1	0.67, 1.81	0,7
**Dexamethasone**					
No	108	56	—	—	
Yes	22	14	1,43	0.79, 2.57	0,23
**Dysgeusia (any grade)**					
No	31	17	—	—	
Yes	98	51	0,89	0.51, 1.54	0,68
**Body mass index [cont.][a]**	129	69	0,98	0.93, 1.03	0,39
**Overweight[a,j]**					
No	65	41	—	—	
Yes	64	28	0,64	0.39, 1.03	0,066

a. Determined prior to first dose of talquetamab. b. Extraosseous disease was defined as the presence of bone-independent/organ-infiltrating disease manifestations or plasma cell leukemia (+/- other extramedullary disease manifestations). Only bone-associated (paramedullary) disease manifestations were not classified as extraosseous disease. c. Based on the latest available cytogenetic findings. d. Chromosome 1q gain (3 copies) or amplification (≥ 4 copies). e. Refractory to at least one immunomodulatory agent, at least one proteasome inhibitor and at least one anti-CD38 monoclonal antibody. f. Refractory to at least two proteasome inhibitors, at least two immunomodulatory agents and at least one anti-CD38 monoclonal antibody. g. Including antibody-drug conjugates, bispecific antibodies and CAR T-cell therapy. h. Including bispecific antibodies and CAR T-cell therapy. i. Combination of ≥ 2 classical cytotoxic agents. j. Body mass index ≥ 25. BCMA, B-cell maturation antigen. BTCE, bispecific T-cell engager. CAR, chimeric antigen receptor. CI, confidence interval. CRS, cytokine release syndrome. ECOG, Eastern Cooperative Oncology Group performance status. eGFR, estimated glomerular filtration rate (CKD-EPI). HR, hazard ratio. ICANS, immune effector cell-associated neurotoxicity. ISS, International Staging System. LDH, lactate dehydrogenase. R-ISS, Revised International Staging System. SCT, stem cell transplant. ULN, upper limit of normal. Q_3, upper/third quartile.

Supp. Table S11 | Univariate Cox regression analysis of duration of response

Characteristic	N	Event N	HR	95% CI	p value
**Eligibility**					
No	40	16	—	—	
Yes	34	6	0,45	0.18, 1.16	0,1
**Age [cont.]**	75	22	0,98	0.93, 1.03	0,43
**Age ≥ 70 years**					
No	54	19	—	—	
Yes	21	3	0,35	0.10, 1.19	0,092
**Gender**					
Male	50	12	—	—	
Female	25	10	1,43	0.61, 3.36	0,41
**ECOG ≥ 2**[a]					
No	60	17	—	—	
Yes	12	4	1,24	0.41, 3.75	0,7
**Extramedullary disease**[a]					
No	43	7	—	—	
Yes	21	11	6,28	2.22, 17.7	**<0.001**
**Extraosseous disease**[a,b]					
No	51	8	—	—	
Yes	13	10	12,3	4.01, 37.5	**<0.001**
**Bone marrow burden ≥ 60%**[a]					
No	20	7	—	—	
Yes	8	1	0,43	0.05, 3.48	0,43
**High risk cytogenetics**[c]					
No	36	9	—	—	
Yes	30	10	1,35	0.55, 3.33	0,51
**+1q**[c,d]					
No	28	8	—	—	
Yes	34	10	0,93	0.36, 2.42	0,88
**ISS stage III**[a]					
No	41	11	—	—	
Yes	22	3	0,48	0.13, 1.74	0,27
**R-ISS stage III**[a]					
No	45	11	—	—	
Yes	15	3	0,72	0.20, 2.67	0,63
**Increased LDH (> ULN)**[a]					
No	44	9	—	—	
Yes	31	13	3,15	1.34, 7.43	**0,009**
**eGFR < 30 ml/min**[a]					
No	68	20	—	—	
Yes	7	2	0,9	0.21, 3.85	0,88
**Prior therapy lines > median (> 6)**					
No	51	11	—	—	
Yes	24	11	1,92	0.83, 4.46	0,13

**Prior allogeneic SCT**					
No	66	19	—	—	
Yes	9	3	1,02	0.30, 3.46	0,98
**Triple-class refractoriness**[e]					
No	12	2	—	—	
Yes	63	20	3,08	0.71, 13.2	0,13
**Penta-drug refractoriness**[f]					
No	45	13	—	—	
Yes	30	9	0,95	0.41, 2.22	0,9
**Prior BCMA-targeted therapy**[g]					
No	35	6	—	—	
Yes	40	16	1,41	0.55, 3.63	0,47
**Prior BTCE therapy**					
No	67	19	—	—	
Yes	8	3	1,19	0.35, 4.07	0,78
**BTCE as last therapy line**					
No	3	2	—	—	
Yes	5	1			
**Response to prior BTCE**					
No	4	2	—	—	
Yes	3	0			
**Time (days) from last BTCE application to first talquetamab dose [cont.]**	7	3			
**Time from prior BTCE therapy to first talquetamab dose > median**					
No	3	1	—	—	
Yes	4	2			
**Time from prior BTCE therapy to first talquetamab dose > Q_3**					
No	4	2	—	—	
Yes	3	1			
**Prior CAR T-cell therapy**					
No	44	10	—	—	
Yes	31	12	1,3	0.54, 3.11	0,56
**Response to prior CAR T-cell therapy**					
No	4	1	—	—	
Yes	25	11	2,85	0.36, 22.7	0,32
**Time (days) from last CAR-T infusion to first talquetamab dose [cont.]**	31	12	1	1.00, 1.00	0,87
**Prior T-cell redirecting immunotherapy**[h]					
No	39	9	—	—	
Yes	36	13	1,11	0.47, 2.65	0,81
**Classical cytotoxic chemotherapy ≤ 60 days prior to first talquetamab dose**					
No	59	15	—	—	
Yes	14	6	1,82	0.70, 4.75	0,22
**Classical cytotoxic polychemotherapy**[i] **≤ 60 days prior to first talquetamab dose**					
No	68	18	—	—	
Yes	7	4	2,7	0.90, 8.09	0,077

**CRS (any grade)**					
No	20	6	—	—	
Yes	55	16	1,06	0.41, 2.70	0,91
**CRS grade ≥ 2**					
No	59	16	—	—	
Yes	16	6	1,17	0.46, 3.00	0,74
**ICANS (any grade)**					
No	72	22	—	—	
Yes	3	0			
**Tocilizumab**					
No	48	13	—	—	
Yes	26	9	1,38	0.59, 3.26	0,46
**Dexamethasone**					
No	62	20	—	—	
Yes	12	2	0,52	0.12, 2.24	0,38
**Dysgeusia (any grade)**					
No	14	2	—	—	
Yes	61	20	1,75	0.41, 7.54	0,45
**Body mass index [cont.]**[a]	73	22	0,99	0.89, 1.10	0,82
**Overweight**[a,j]					
No	33	13	—	—	
Yes	40	9	0,7	0.30, 1.65	0,41

a. Determined prior to first dose of talquetamab. b. Extraosseous disease was defined as the presence of bone-independent/organ-infiltrating disease manifestations or plasma cell leukemia (+/- other extramedullary disease manifestations). Only bone-associated (paramedullary) disease manifestations were not classified as extraosseous disease. c. Based on the latest available cytogenetic findings. d. Chromosome 1q gain (3 copies) or amplification (≥ 4 copies). e. Refractory to at least one immunomodulatory agent, at least one proteasome inhibitor and at least one anti-CD38 monoclonal antibody. f. Refractory to at least two proteasome inhibitors, at least two immunomodulatory agents and at least one anti-CD38 monoclonal antibody. g. Including antibody-drug conjugates, bispecific antibodies and CAR T-cell therapy. h. Including bispecific antibodies and CAR T-cell therapy. i. Combination of ≥ 2 classical cytotoxic agents. j. Body mass index ≥ 25. BCMA, B-cell maturation antigen. BTCE, bispecific T-cell engager. CAR, chimeric antigen receptor. CI, confidence interval. CRS, cytokine release syndrome. ECOG, Eastern Cooperative Oncology Group performance status. eGFR, estimated glomerular filtration rate (CKD-EPI). HR, hazard ratio. ICANS, immune effector cell-associated neurotoxicity. ISS, International Staging System. LDH, lactate dehydrogenase. R-ISS, Revised International Staging System. SCT, stem cell transplant. ULN, upper limit of normal. Q_3, upper/third quartile.

Supp. Table S12 │ Univariate Cox regression analysis of overall survival

Characteristic	N	Event N	HR	95% CI	p value
**Eligibility**					
No	73	27	—	—	
Yes	54	13	0,58	0.30, 1.12	0,1
**Age [cont.]**	131	41	1	0.97, 1.03	0,81
**Age ≥ 70 years**					
No	96	28	—	—	
Yes	35	13	1,16	0.60, 2.25	0,66
**Gender**					
Male	92	31	—	—	
Female	39	10	0,73	0.36, 1.49	0,39
**ECOG ≥ 2**[a]					
No	100	27	—	—	
Yes	27	14	2,41	1.26, 4.60	**0,008**
**Extramedullary disease**[a]					
No	63	15	—	—	
Yes	54	22	1,89	0.98, 3.65	0,058
**Extraosseous disease**[a,b]					
No	77	18	—	—	
Yes	40	19	2,37	1.24, 4.52	**0,009**
**Bone marrow burden ≥ 60%**[a]					
No	31	10	—	—	
Yes	15	7	2,63	0.97, 7.15	0,058
**High risk cytogenetics**[c]					
No	60	20	—	—	
Yes	53	16	1,01	0.52, 1.96	0,97
**+1q**[c,d]					
No	53	19	—	—	
Yes	53	17	0,8	0.42, 1.54	0,5
**ISS stage III**[a]					
No	64	10	—	—	
Yes	44	18	3,06	1.41, 6.63	**0,005**
**R-ISS stage III**[a]					
No	73	14	—	—	
Yes	31	13	2,32	1.09, 4.94	**0,029**
**Increased LDH (> ULN)**[a]					
No	71	15	—	—	
Yes	60	26	2,62	1.39, 4.96	**0,003**
**eGFR < 30 ml/min**[a]					
No	119	36	—	—	
Yes	12	5	1,64	0.64, 4.18	0,3
**Prior therapy lines > median (> 6)**					
No	85	21	—	—	
Yes	46	20	1,94	1.05, 3.58	**0,035**

**Prior allogeneic SCT**					
No	115	34	—	—	
Yes	16	7	1,47	0.65, 3.34	0,36
**Triple-class refractoriness**[e]					
No	19	7	—	—	
Yes	112	34	0,96	0.42, 2.17	0,92
**Penta-drug refractoriness**[f]					
No	68	19	—	—	
Yes	61	22	1,43	0.78, 2.65	0,25
**Prior BCMA-targeted therapy**[g]					
No	61	15	—	—	
Yes	70	26	1,52	0.80, 2.87	0,2
**Prior BTCE therapy**					
No	104	28	—	—	
Yes	27	13	2,01	1.04, 3.88	**0,038**
**BTCE as last therapy line**					
No	10	4	—	—	
Yes	17	9	1,95	0.59, 6.47	0,27
**Response to prior BTCE**					
No	13	9	—	—	
Yes	11	3	0,32	0.09, 1.19	0,089
**Time (days) from last BTCE application to first talquetamab dose [cont.]**	26	13	1	0.99, 1.00	0,19
**Time from prior BTCE therapy to first talquetamab dose > median**					
No	14	9	—	—	
Yes	12	4	0,31	0.09, 1.03	0,055
**Time from prior BTCE therapy to first talquetamab dose > Q_3**					
No	21	12	—	—	
Yes	5	1	0,23	0.03, 1.79	0,16
**Prior CAR T-cell therapy**					
No	84	23	—	—	
Yes	47	18	1,4	0.76, 2.60	0,28
**Response to prior CAR T-cell therapy**					
No	6	3	—	—	
Yes	37	14	0,71	0.20, 2.53	0,6
**Time (days) from last CAR-T infusion to first talquetamab dose [cont.]**	46	18	1	1.00, 1.00	0,74
**Prior T-cell redirecting immunotherapy**[h]					
No	66	16	—	—	
Yes	65	25	1,64	0.87, 3.06	0,12
**Classical cytotoxic chemotherapy ≤ 60 days prior to first talquetamab dose**					
No	97	29	—	—	
Yes	32	11	1,1	0.55, 2.20	0,79
**Classical cytotoxic polychemotherapy**[i] **≤ 60 days prior to first talquetamab dose**					
No	116	35	—	—	
Yes	15	6	1,18	0.50, 2.82	0,7

**CRS (any grade)**					
No	41	14	—	—	
Yes	90	27	0,9	0.47, 1.73	0,76
**CRS grade ≥ 2**					
No	104	33	—	—	
Yes	27	8	0,96	0.44, 2.08	0,91
**ICANS (any grade)**					
No	122	33	—	—	
Yes	8	7	8,22	3.47, 19.5	**<0.001**
**Tocilizumab**					
No	87	26	—	—	
Yes	43	15	1,23	0.65, 2.33	0,52
**Dexamethasone**					
No	108	33	—	—	
Yes	22	8	1,35	0.62, 2.94	0,45
**Dysgeusia (any grade)**					
No	31	13	—	—	
Yes	98	27	0,46	0.24, 0.90	**0,022**
**Body mass index [cont.]**[a]	129	40	0,97	0.91, 1.04	0,44
**Overweight**[a,j]					
No	65	24	—	—	
Yes	64	16	0,6	0.32, 1.14	0,12

a. Determined prior to first dose of talquetamab. b. Extraosseous disease was defined as the presence of bone-independent/organ-infiltrating disease manifestations or plasma cell leukemia (+/- other extramedullary disease manifestations). Only bone-associated (paramedullary) disease manifestations were not classified as extraosseous disease. c. Based on the latest available cytogenetic findings. d. Chromosome 1q gain (3 copies) or amplification (≥ 4 copies). e. Refractory to at least one immunomodulatory agent, at least one proteasome inhibitor and at least one anti-CD38 monoclonal antibody. f. Refractory to at least two proteasome inhibitors, at least two immunomodulatory agents and at least one anti-CD38 monoclonal antibody. g. Including antibody-drug conjugates, bispecific antibodies and CAR T-cell therapy. h. Including bispecific antibodies and CAR T-cell therapy. i. Combination of ≥ 2 classical cytotoxic agents. j. Body mass index ≥ 25. BCMA, B-cell maturation antigen. BTCE, bispecific T-cell engager. CAR, chimeric antigen receptor. CI, confidence interval. CRS, cytokine release syndrome. ECOG, Eastern Cooperative Oncology Group performance status. eGFR, estimated glomerular filtration rate (CKD-EPI). HR, hazard ratio. ICANS, immune effector cell-associated neurotoxicity. ISS, International Staging System. LDH, lactate dehydrogenase. R-ISS, Revised International Staging System. SCT, stem cell transplant. ULN, upper limit of normal. Q_3, upper/third quartile.
